# Web-Based Risk Prediction Tool for an Individual's Risk of HIV and Sexually Transmitted Infections Using Machine Learning Algorithms: Development and External Validation Study

**DOI:** 10.2196/37850

**Published:** 2022-08-25

**Authors:** Xianglong Xu, Zhen Yu, Zongyuan Ge, Eric P F Chow, Yining Bao, Jason J Ong, Wei Li, Jinrong Wu, Christopher K Fairley, Lei Zhang

**Affiliations:** 1 Melbourne Sexual Health Centre Alfred Health Melbourne Australia; 2 Central Clinical School Faculty of Medicine, Nursing and Health Sciences Monash University Melbourne Australia; 3 China Australia Joint Research Center for Infectious Diseases Xi'an Jiaotong University Health Science Centre Xi'an China; 4 Monash e-Research Centre Faculty of Engineering, Airdoc Research Nvidia AI Technology Research Centre, Monash University Melbourne Australia; 5 Centre for Epidemiology and Biostatistics Melbourne School of Population and Global Health University of Melbourne Melbourne Australia; 6 School of Public Health Southeast University Nanjing China; 7 Research Centre for Data Analytics and Cognition La Trobe University Melbourne Australia

**Keywords:** HIV, sexually transmitted infections, syphilis, gonorrhea, chlamydia, sexual health, sexual transmission, sexually transmitted, prediction, web-based, risk assessment, machine learning, model, algorithm, predictive, risk, development, validation

## Abstract

**Background:**

HIV and sexually transmitted infections (STIs) are major global public health concerns. Over 1 million curable STIs occur every day among people aged 15 years to 49 years worldwide. Insufficient testing or screening substantially impedes the elimination of HIV and STI transmission.

**Objective:**

The aim of our study was to develop an HIV and STI risk prediction tool using machine learning algorithms.

**Methods:**

We used clinic consultations that tested for HIV and STIs at the Melbourne Sexual Health Centre between March 2, 2015, and December 31, 2018, as the development data set (training and testing data set). We also used 2 external validation data sets, including data from 2019 as external “validation data 1” and data from January 2020 and January 2021 as external “validation data 2.” We developed 34 machine learning models to assess the risk of acquiring HIV, syphilis, gonorrhea, and chlamydia. We created an online tool to generate an individual’s risk of HIV or an STI.

**Results:**

The important predictors for HIV and STI risk were gender, age, men who reported having sex with men, number of casual sexual partners, and condom use. Our machine learning–based risk prediction tool, named MySTIRisk, performed at an acceptable or excellent level on testing data sets (area under the curve [AUC] for HIV=0.78; AUC for syphilis=0.84; AUC for gonorrhea=0.78; AUC for chlamydia=0.70) and had stable performance on both external validation data from 2019 (AUC for HIV=0.79; AUC for syphilis=0.85; AUC for gonorrhea=0.81; AUC for chlamydia=0.69) and data from 2020-2021 (AUC for HIV=0.71; AUC for syphilis=0.84; AUC for gonorrhea=0.79; AUC for chlamydia=0.69).

**Conclusions:**

Our web-based risk prediction tool could accurately predict the risk of HIV and STIs for clinic attendees using simple self-reported questions. MySTIRisk could serve as an HIV and STI screening tool on clinic websites or digital health platforms to encourage individuals at risk of HIV or an STI to be tested or start HIV pre-exposure prophylaxis. The public can use this tool to assess their risk and then decide if they would attend a clinic for testing. Clinicians or public health workers can use this tool to identify high-risk individuals for further interventions.

## Introduction

HIV and sexually transmitted infections (STIs) are major global public health concerns [1,2]. The World Health Organization (WHO) estimated that over 1 million curable STIs occur every day among people aged 15 years to 49 years worldwide [3]. An estimated 29,090 people have been infected with HIV in Australia as of the end of 2020, with an HIV prevalence rate of 0.14% among people over 15 years old [4]. The estimated undiagnosed HIV infection rate among all people living with HIV in Australia was about 9% in 2020 [4]. Gonorrhea, chlamydia, and early syphilis can be asymptomatic. There were large increases in STIs in Australia between 2013 and 2017. The notification rates of STIs for chlamydia increased from 302.2/100,000 to 394.9/100,000 in men and from 430.7/100,000 to 441.8/100,000 in women, gonorrhea increased from 91.1/100,000 to 174.2/100,000 in men and from 39.6/100,000 to 61.8/100,000 in women, and syphilis increased from 12.3/100,000 to 31.1/100,000 in men and from 1.4/100,000 to 5.5/100,000 in women [5]. In addition, STIs account for a large health and economic burden in limited-income countries [6].

In response to the rising rates of STIs, the WHO proposed the “Global health sector strategy on Sexually Transmitted Infections, 2016-2021,” which aimed to end STI epidemics as public health concerns by 2030. This specifically includes a 90% reduction in gonorrhea incidence globally from the 2018 global baseline and achieving a rate of ≤50 congenital syphilis cases per 100,000 live births in 80% of countries [7]. In 2018, the United Nations proposed “The 2030 Agenda for Sustainable Development,” which called for an end to the AIDS epidemic by 2030 [8]. Key to the effective control of these infections is accessible health care and, in particular, frequent testing because treated infections rapidly become noninfectious [2]. Screening of asymptomatic individuals is important for diagnosis, treatment, prevention, and control of HIV and STIs [9]. Barriers to testing include misjudgment of an individual's HIV and STI risk, limited availability of testing, and high cost of testing [10]. Therefore, developing innovative tools will help individuals accurately judge their risk of HIV and STIs, hence increasing screening in high-risk individuals.

An easily accessible and user-friendly tool that accurately identifies an individual's risk of infection could form part of a web-based risk prediction program and play a role in risk prediction and personalized risk management [11]. Providing the public with risk prediction tools to assist them in estimating the risk of HIV and STIs may encourage those individuals at high risk to test more regularly. A previous study showed that increased risk perceptions were associated with greater STI health care use (eg, testing) [12]. An HIV and STI risk prediction tool may increase risk perceptions and motivate individuals to seek HIV and STI testing or treatment. Another review study suggested that web-based screening apps can effectively increase the uptake of health screening in the general population [13]. However, there is no web-based tool we could identify that provides users with an individual’s current quantitative risk of HIV and STIs (gonorrhea, chlamydia, and syphilis) using self-reported questions.

A number of mathematical techniques can be used to generate an individual’s risk of HIV and STIs. Logistic regression has limitations in predictive analysis that uses complex and big data. Logistic regression methods require strong assumptions and cannot easily deal with nonlinear relationships, interactions, and multicollinearity [14,15]. In contrast, nonlinear machine learning approaches can address these limitations and have numerous advantages (eg, capturing nonlinear relationships and interactions) in predictive analysis using big data [16]. Machine learning also can identify rare health outcomes with high accuracy [17]. Ensemble learning is also a machine learning approach that combines multiple machine learning algorithms to improve the model's performance [18]. 

Despite the advantages of machine learning approaches, there is an absence of individual risk prediction tools for HIV and STI risk using machine learning models. Existing studies using machine learning algorithms to predict HIV and STI acquisition mainly focus on HIV [19-30], and few focus on STIs [19,21,31]. Of these HIV prediction studies, 4 studies focused on high-risk individuals (such as men who have sex with men [MSM] [20,21,24,29]), 2 studies used imaging or clinical text data [22,30], 4 studies used more than 40 predictors [23,26-28], and 2 studies assessed future but not current HIV prediction [19,25]. Of the STI prediction studies, 1 study was conducted with MSM [21], and the other 2 studies focused on future STI prediction [19,31]. These studies also found that nonlinear machine learning models (eg, random forest [RF], gradient boosting machine [GBM], and neural networks) performed better than logistic regression in HIV and STI prediction [19,21,24,31]. These published studies highlight a lack of machine learning models that use simple self-reported questions, predict both the risk of HIV and STIs, and can be used by both men and women. Therefore, to address the current lack of studies that predict the risk of both STIs and HIV, particularly in lower-risk heterosexual individuals, we aimed to use a stacking ensemble learning framework and self-reported questions to predict HIV and 3 common STIs (gonorrhea, chlamydia, and syphilis) in both men and women and a subsequent web-based HIV and STI risk prediction tool.

## Methods

### Study Population

The Melbourne Sexual Health Centre (MSHC) is the largest public sexual health center in Victoria, Australia and offers free HIV and STI testing and management [32]. At the MSHC, individuals' demographic information and sexual practices are recorded using a computer-assisted self-interview (CASI) at each visit, at least 3 months apart [33]. We used clinical consultation data from the electronic health record (EHR) at MSHC to develop and validate the risk prediction model. We chose March 2, 2015, as the commencement date because this date was when we adopted a new testing platform for gonorrhea and chlamydia (Aptima Combo, Hologic, Marlborough, MA). Our study data included men and women aged 18 years and older who was tested for HIV or an STI at the MSHC between March 2, 2015, and January 29, 2021. We excluded transgender people and individuals aged younger than 18 years.

We used data from March 2, 2015, to December 31, 2018, as the development data set (training and testing data set). The HIV study data set included training and testing data (88,642 consultations). The syphilis, gonorrhea, and chlamydia study data sets had 92,291, 97,473, and 115,845 consultations, respectively.

We used temporal validation as the external validation to evaluate the transportability and generalizability of our risk prediction models. The COVID-19 epidemic may potentially have changed the demographics of those who attend the MSHC [34]. We performed 2 temporal validations to validate our models further and reduce the possible bias caused by COVID-19. The 2 external validation data sets included data from 2019 as external “validation data 1” and data from January 2020 and January 2021 as external “validation data 2.” For HIV, the first external validation data set contained 28,875 consultations, and the second external validation data set contained 18,052 consultations. For syphilis, the first external validation data set contained 30,302 consultations, and the second external validation data set contained 19,150 consultations. For gonorrhea, the first external validation data set contained 36,805 consultations, and the second external validation data set contained 22,886 consultations. For chlamydia, the first external validation data set contained 36,393 consultations, and the second external validation data set contained 22,615 consultations.

### Ethical Approval

Ethical approval was granted by the Alfred Hospital Ethics Committee, Melbourne, Australia (project number: 124/18). All methods were carried out following relevant guidelines and regulations of the Alfred Hospital Ethics Committee. As this was a retrospective study involving minimal risk to the privacy of the study participants, the need for informed consent was waived by the Alfred Hospital Ethics Committee. All identifying details of the study participants were removed before any computational analysis.

### Predictors

The data fields we selected for inclusion as predictors were informed by literature review, expert opinion, and prior work [21]. The predictors were self-reported questions from the EHR, including demographics, sexual practices, STI history, and STI contact history (summarized in Table 1 and Tables S1-S5 in Multimedia Appendix 1).

**Table 1:** Characteristics of clinic consultations in the training and testing data set.

Variables		HIV (n=88,642 consultations)	Syphilis (n=92,291 consultations)	Gonorrhea (n=97,473 consultations)	Chlamydia (n=115,845 consultations)
**Gender, n (%)**					
	Female	26,651 (30.1)	27,134 (29.4)	31,282 (32.1)	38,548 (33.3)
	Male	61,991 (69.9)	65,157 (70.6)	66,191 (67.9)	77,297 (66.7)
Age at consultation (years), median (IQR)		29.0 (24.0-35.0)	29.0 (25.0-35.0)	28.0 (24.0-35.0)	28.0 (24.0-34.0)
**Country of birth, n (%)**					
	Australia	39,148 (44.2)	40,990 (44.4)	43,881 (45.0)	51,162 (44.2)
	Overseas	46,003 (51.9)	47,670 (51.7)	49,835 (51.1)	60,272 (52.0)
	Missing	3491 (3.9)	3631 (3.9)	3757 (3.9)	4411 (3.8)
**STI^a^ symptoms, n (%)**					
	No	56,175 (63.4)	57,413 (62.2)	54,595 (56.0)	68,584 (59.2)
	Yes	25,067 (28.3)	27,150 (29.4)	34,751 (35.7)	38,930 (33.6)
	Missing	7383 (8.3)	7728 (8.4)	8127 (8.3)	8331 (7.2)
**Men who have sex with men, n (%)**					
	Not applicable (female)	26,651 (30.1)	27,134 (29.4)	31,282 (32.1)	38,548 (33.3)
	No	16,508 (18.6)	17,089 (18.5)	15,245 (15.6)	26,975 (23.3)
	Yes	45,483 (51.3)	48,068 (52.1)	50,946 (52.3)	50,322 (43.4)

^a^STI: sexually transmitted infection**.**

### Measurement of Outcomes

HIV infection was defined as a new diagnosis of HIV based on serology. Syphilis infection was defined as a new diagnosis of early syphilis (primary, secondary, and early latent [<2 years]) using a blood test or nucleic amplification test (NAAT). Gonorrhea infection was defined as a new diagnosis of gonorrhea using culture or NAAT at any anatomical site. In the clinic, gonorrhea testing initially occurs with NAAT, and culture is mostly used after a positive NAAT. Chlamydia infection was defined as a new diagnosis using NAAT at any anatomical site. Our previous publications report the diagnostic methods in detail [19,21].

### Risk Assessment Model Development

**Figure 1:**
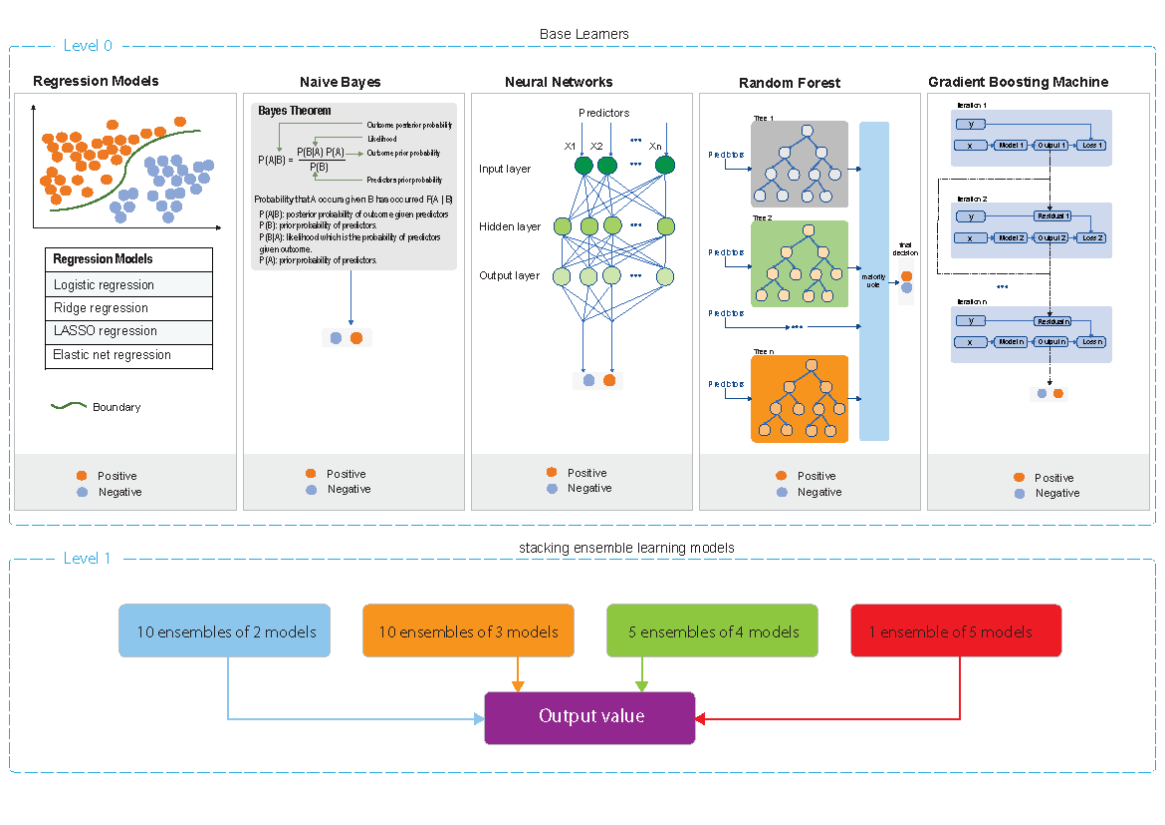
Development of machine learning algorithms. The architecture of the gradient boosting machine was adapted from Feng et al [35]. LASSO: least absolute shrinkage and selection operator.

We developed 34 machine learning models to assess the risk of acquiring HIV, syphilis, gonorrhea, and chlamydia (details in Figure 1).

#### Base Learner

Logistic regression has been widely used to predict the risk of incident STIs and HIV [36,37]. GBM uses boosting based on decision trees by adjusting the parameters to minimize a loss function and determine the optimal point with the smallest error [38]. RF comprises an ensemble of decision trees using bootstrap aggregation and randomization of predictors to achieve a high degree of predictive accuracy [39]. Naive Bayes (NB) is simple, has high accuracy and speed in large databases, and has been widely used for disease classification [40]. Deep learning (DL) has effectively solved many medical problems and utilizes a hierarchical level of an artificial neural network to perform the classification process [41].

We first established 4 regression models, including logistic regression, ridge regression, least absolute shrinkage and selection operator (LASSO) regression, and elastic net regression (ENR). Based on the preliminary results of the 4 regression analyses, we found that ENR was better than the other 3 regression analyses (details in Multimedia Appendix 1). Considering our previous machine learning study among MSM [21] and the advantages of NB (eg, high accuracy and speed in large databases), we developed 5 base models, including the aforementioned ENR, NB, DL (neural networks), RF, and GBM.

#### Stacking Ensemble Learning

Stacking ensemble learning is an ensemble learning method that trains a new model based on the combined predictions of 2 (or more) previous machine learning models. Stacking ensemble learning often performs better than individual machine learning techniques [42]. We systematically established 26 ensemble learning models by combining the aforementioned 5 base models to improve the performance of predicting HIV and STIs. Details are in Multimedia Appendix 1 (summarized in Table S6).

#### Machine Learning Training Techniques

Our models used a one-hot encoding scheme for data classification. We did not impute missing data but created a binary feature vector indicating missing values. The data were considered “imbalanced” given that each of the 4 infections was <10%. Imbalanced data may cause either overfitted or underperformed predictive results [43]. We used 5 x 10 (5 outer folds, 10 inner folds) nested cross-validation (CV) for model selection and training [21,44]. The outer 5-fold CV was used to address the selection bias caused by using a single data set. The inner 10-fold CV was used on the training data set to perform the hyperparameter tuning of machine learning models. We used the area under the curve (AUC) to select the best model. An AUC of 0.7 to 0.8 is considered acceptable, 0.8 to 0.9 is considered excellent, and >0.9 is considered outstanding [45]. Machine learning models were built using the *h2o* package (version 3.32.1.2) in R software (3.6.1 and R studio 1.2.5019).

### Estimating the Risk of HIV and STIs

Our machine learning models predicted the probability of HIV or an STI with a normalized distribution between values 0 and 1. The model-predicted probability was calibrated to the actual prevalence level of HIV and STIs. We used a logistic function to provide a fitting curve for each model-predicted probability and infection prevalence. We regarded the estimated infection prevalence as the “calibrated risk” of infection and presented it in the risk report. We used MATLAB R2019a (MathWorks, Natick, MA) to calibrate the model-predicted probability to the actual prevalence level. The method is described in detail in our previous paper [19]. We classified the calibrated risk of HIV or an STI into 3 risk levels: HIV (low, <0.1%; medium, 0.1%-1.0%; and high, >1.0%), syphilis (low, <0.2%; medium, 0.2%-5.0%; and high, >5.0%), gonorrhea (low, <0.1%; medium, 0.1%-1.0%; and high, ≥1.0%), and chlamydia (low, <2.0%; medium, 2.0%-15.0%; and high, >15.0%).

### Establishment of a HIV and STI Risk Prediction Tool

To investigate the effect of predictors, we used the best base machine learning model to calculate the variable importance for HIV, syphilis, gonorrhea, and chlamydia infection. We identified and selected predictors that accounted for more than 80.0% of the overall model performance for each infection. We retrained, retested, and revalidated the best performing model based on these predictors. We compared the AUC, sensitivity, and specificity to re-evaluate the model performance with the shortlisted predictors. We also used the AUC to evaluate the change in performance in the best machine learning model before and after predictor shortlisting (details in Multimedia Appendix 1). We formed a new questionnaire by pooling the important predictors to develop a web-based tool for HIV and STI risk prediction.

## Results

### Characteristics of the Study Data

Our training and testing data included 216 (0.2% of 88,642 consultations) HIV infections, 787 (1.9% of 92,291 consultations) syphilis infections, 7581 (7.8% of 97,473 consultations) gonorrhea infections, and 10,217 (8.8% of 115,845 consultations) chlamydia infections. The proportion of each of the 4 infection data sets that was men was between 66.7% (77,297/115,845) and 70.6% (65,157/92,291). Further details are provided in Table 1 and Table S1 in Multimedia Appendix 1. The characteristics of the external validation data are shown in Tables S2-S5 in Multimedia Appendix 1.

### Selecting the Best ML Model for the HIV and STI Risk Prediction Tool

Our results demonstrated that the ensemble learning models performed better than individual machine learning models. Of all 34 models, our best model (ensemble ENR+GBM+RF) provided acceptable or excellent performance on testing data for predicting HIV (AUC=0.78), syphilis (AUC=0.84), gonorrhea (AUC=0.78), and chlamydia (AUC=0.70; Figures S1-S3 in Multimedia Appendix 1). Details on the testing data analysis are provided in Tables S7-S22 in Multimedia Appendix 1. Our external validation results showed very comparable AUCs (0.69-0.85) to the testing data analysis. Details on the external validation analysis are provided in Tables S7-S22 in Multimedia Appendix 1.

### Selecting the Most Important Predictors for the HIV and STI Risk Prediction Tool

**Figure 2:**
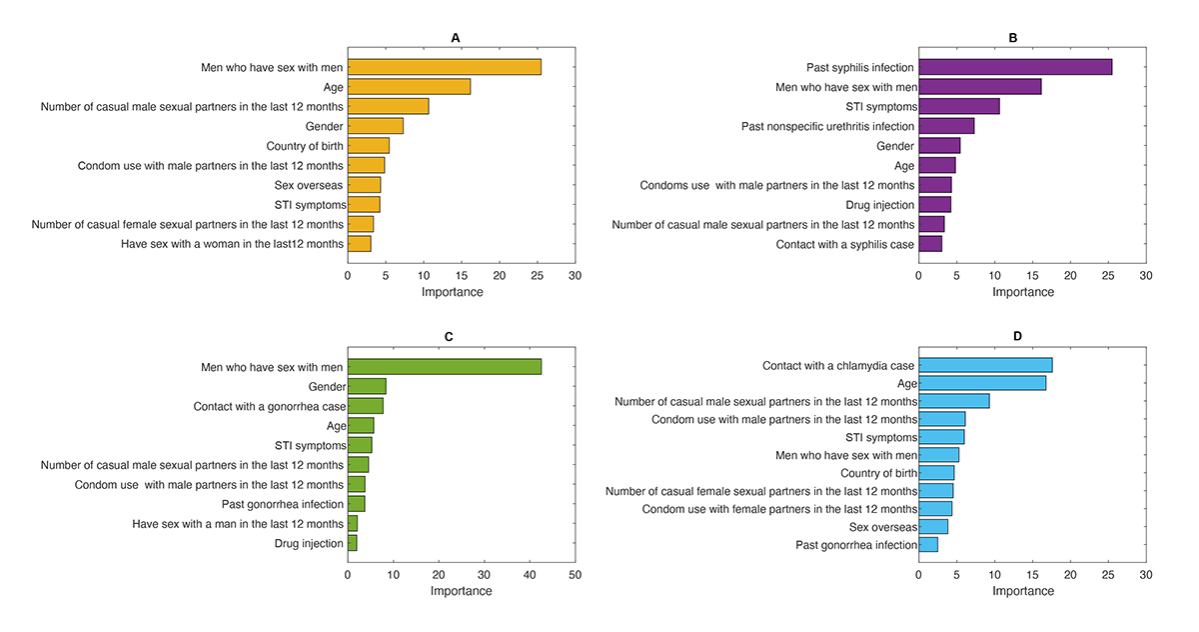
Importance of the top 10 predictors in the prediction of HIV or sexually transmission infections (STIs) using a gradient boosting machine, for detecting (A) HIV, (B) syphilis, (C) gonorrhea, and (D) chlamydia.

The top 10 predictors for each of the 4 infections accounted for >80.0% of the overall HIV and STI model performance. These predictors included gender, presence of STI symptoms, MSM, age, country of birth, having sex with a man in the last 12 months, the number of casual male sexual partners in the last 12 months, condom use with male partners in the last 12 months, the number of casual female sexual partners in the last 12 months, drug injection in the last 12 months, sex overseas in the last 12 months, past gonorrhea infection, past nonspecific urethritis infection, past syphilis infection, contact with a gonorrhea case, contact with a chlamydia case, and contact with a syphilis case (Figure 2). We formed the final HIV and STI risk prediction questionnaire with the top 10 predictors for each infection.

### Establishment and Evaluation of the HIV and STI Risk Prediction Tool, MySTIRisk

**Figure 3:**
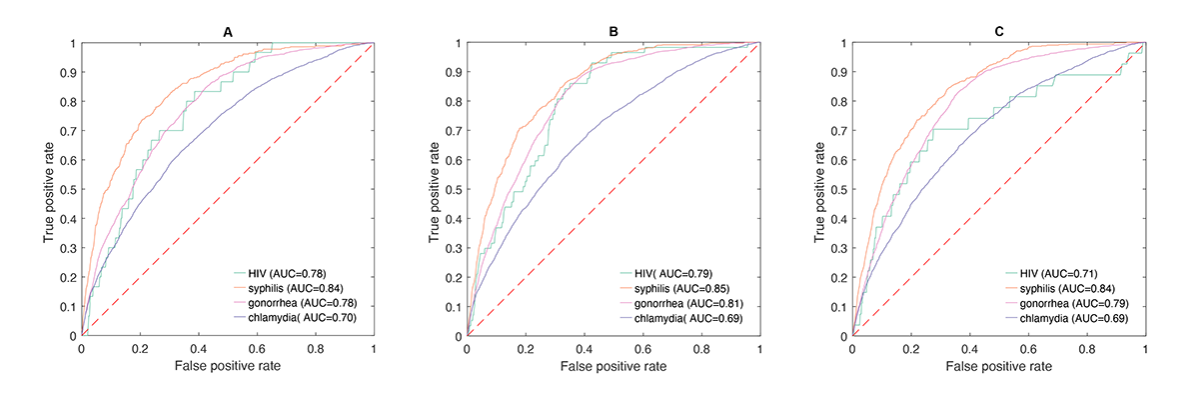
Receiver operating characteristic curve performance of the HIV and sexually transmitted infection (STI) risk prediction tool on (A) testing data analysis from 2015-2018, (B) external data validation analysis from 2019, and (C) external data validation analysis from 2020-2021. AUC: area under the curve.

Based on the selected most important predictors and the best model (ensemble ENR+GBM+RF), we built a HIV and STI risk prediction tool, named *MySTIRisk*. We examined *MySTIRisk* and demonstrated its performance on testing to be acceptable or excellent (AUC for HIV=0.78; AUC for syphilis=0.84; AUC for gonorrhea=0.78; AUC for chlamydia=0.70), similar to its original model based on predictors. Our risk prediction tool obtained stable performance on external validation data from 2019 (AUC for HIV=0.79; AUC for syphilis=0.85; AUC for gonorrhea=0.81; AUC for chlamydia=0.69). Our risk prediction tool also achieved stable performance on external validation data from 2020-2021 (AUC for HIV=0.71; AUC for syphilis=0.84; AUC for gonorrhea=0.79; AUC for chlamydia=0.69; Figure 3 and Tables S23-S26 in Multimedia Appendix 1). Using the selected predictors, our risk prediction tool showed comparable AUCs to the best machine learning model using all predictors (Table S27 in Multimedia Appendix 1).

**Figure 4:**
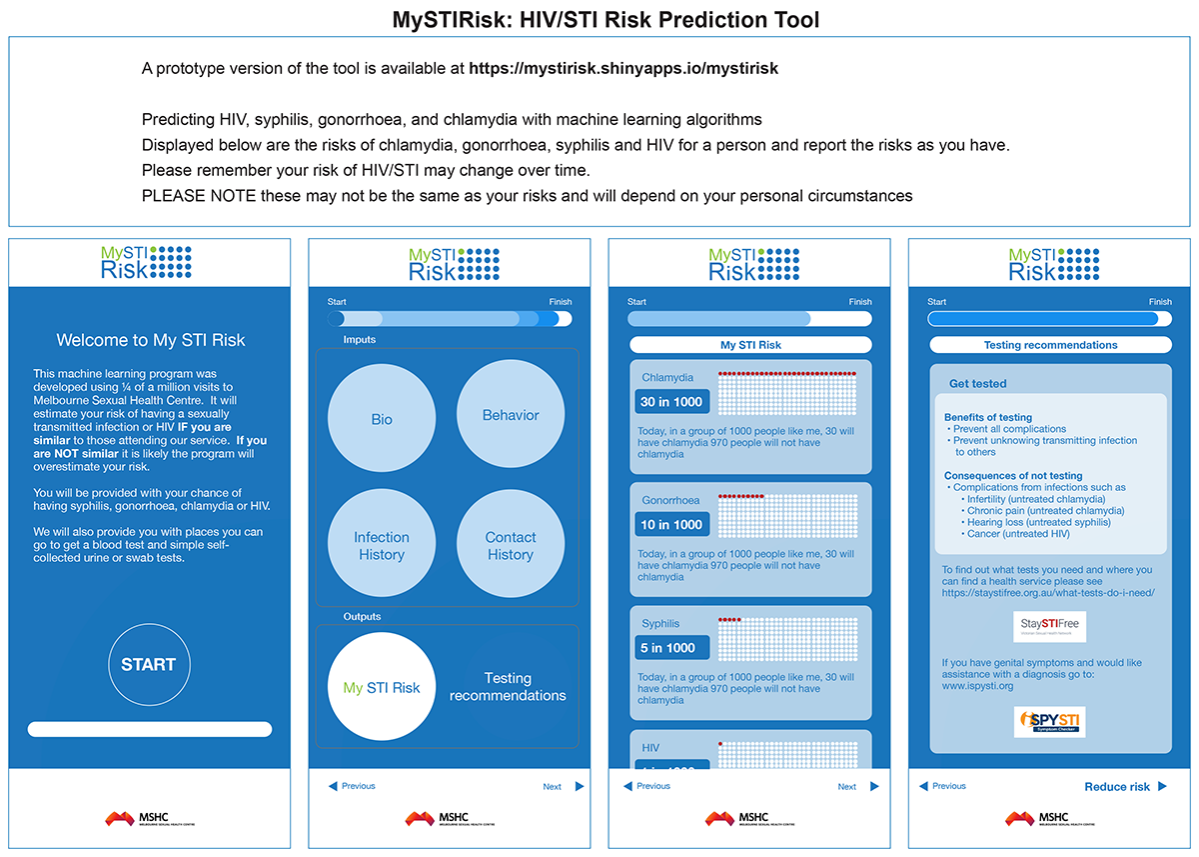
Graphical user interface elements of the HIV and sexually transmitted infection (STI) risk prediction tool, called MySTIRisk. A prototype version of the tool is available at [48]. Machine learning algorithms are used to predict a person’s risk of chlamydia, gonorrhea, syphilis, and HIV.

To estimate the risk of HIV or an STI, we fitted the data using a logistic function to provide a fitting curve for each model-predicted probability and infection prevalence (Figures S4-S7 in Multimedia Appendix 1). Then, a prototype version of the tool was created with R Shiny [46,47] to allow for individual input and HIV and STI risk computation. A prototype version of the tool is available online [48]. The graphical user interface elements of the tool are summarized in Figure 4. The web application collects individual characteristics, processes the collected characteristics, loads the trained machine learning models, calculates a quantitative HIV and STI risk, and displays the results of the risk and recommendations. The web application’s input was designed using previous successful websites or internal CASI questionnaires (60,000 entries a year) that operate at MSHC and used individual characteristic data, including demographics, sexual practices, STI history, and STI contact history. The web application's output includes HIV and STI risk prediction results and recommendations that were developed in consultation with Professor Jon Emery at the University of Melbourne, who is an expert in the communication of risk (see the Acknowledgments section). We acknowledge that this is a prototype and that further development will take place in optimizing this output for accurate risk communication.

These are examples of the HIV and STI risk prediction results:

Your HIV risk is about 2/1000. In a group of 1000 people like me, 2 will have HIV. 998 people will not have HIV.

Your syphilis risk is about 10/1000. In a group of 1000 people like me, 10 will have syphilis. 990 people will not have syphilis.

Your gonorrhea risk is about 30/1000. In a group of 1000 people like me, 30 will have gonorrhea. 970 people will not have gonorrhea.

Your chlamydia risk is about 50/1000. In a group of 1000 people like me, 50 will have chlamydia. 950 people will not have chlamydia.

The following examples describe testing recommendations:

- Benefits of testing: Prevent all complications and prevent unknowingly transmitting infection to others.
- Consequences of not testing: Complications from infections such as infertility (untreated chlamydia), chronic pain (untreated chlamydia), hearing loss (untreated syphilis), and cancer (untreated HIV).

## Discussion

### Principal Findings

This is the first web-based risk prediction tool based on machine learning algorithms and self-reported data to accurately identify HIV and syphilis, gonorrhea, and chlamydia infection in men and women and was stable on external validation. Our findings showed that machine learning algorithms could predict HIV and STIs in clinic attendees. Our results also showed that stacking ensemble learning algorithms perform better than individual machine learning models to predict HIV and STIs. We then developed a web-based application to provide an immediate and individualized assessment for the risk of a positive diagnosis of HIV and 3 STIs. Our application could be a part of clinic websites or digital health platforms to identify individuals with a higher risk of HIV and STIs or potential candidates for HIV pre-exposure prophylaxis (PrEP). Further validation studies in other countries can assess the usefulness of this risk prediction tool, which helps reduce HIV and STI incidence and the cost of HIV and STI screening, which requires expensive equipment and specialized expertise.

### Comparison With Prior Work

Our results showed that nonlinear machine learning algorithms provided better performance than the conventional logistic regression for predicting HIV and STIs in men and women. Our findings are consistent with the results of previous machine learning predictive models for HIV and STIs [19,21,24,31]. Bao et al [21] showed that a GBM model performed better than logistic regression in MSM. Our study suggests that nonlinear machine learning models (eg, GBM, RF) could provide better performance than conventional logistic regression even without ensemble learning.

Our results showed that the stacking ensemble machine learning techniques outperform individual machine learning models. We systematically developed and tested 34 machine learning models and found that stacking ensemble learning technology outperformed individual machine learning models [18]. Previous studies have used ensemble learning models to predict an individual's HIV risk [19,25]; however, no study has looked at the risk of gonorrhea, chlamydia, or syphilis using ensemble learning models. The only study we could identify was one that had predicted the risk of a repeat STI with ensemble learning. Elder et al [31] showed that an ensemble of models could perform better for 2 or more repeat STIs within 730 days of follow-up than the individual classifiers (AUC=0.76). Our results found that stacking ensemble techniques could also be applied to enhance the performance of HIV prediction. The AUC of our ensemble HIV model (AUC=0.78, 95% CI 0.74-0.83) was higher than that in a similar study in Kenya and Uganda for HIV risk prediction (AUC=0.73, 95% CI 0.71-0.76) [25]. We also found that the combinations of more individual machine learning models do not necessarily lead to a better stacking ensemble model. For example, in our study, the stacking ensemble learning of 4 models for syphilis was not higher than a stacking ensemble learning of 3 models. We also found that a better performing stacking ensemble model always included GBM. The findings of our stacking ensemble learning strategies may have implications for future stacking ensemble learning frameworks.

Our models have several strengths compared with previous machine learning models for predicting HIV and STIs. First, our predictive models were not limited to high-risk groups (such as MSM). HIV and STI risk prediction models have been published previously but mainly for high-risk individuals, such as MSM [20,21,24,29]. Our models could predict HIV and STI acquisition in both men and women, including homosexual and heterosexual individuals. Second, our predictive models only used self-reported and simple questions to develop models. Previously published studies used numerous predictors for their models [23,26-28]. Third, we systematically developed 26 ensemble models. In our study, we tested all possible combinations of 5 base models. The final strength of our research is that we performed 2 external validation analyses of each model.

We were unable to locate any web-based, publicly available tool to quantify STI risk. We identified some available web-based HIV prediction tools, such as the “HIV risk prediction tool” [49], “HIV/AIDS Risk Calculator” [50], and “Online Risk Assessment” [51]. We also identified some available web-based STI prediction tools, such as “Find out if you need to get tested for an STD” [52], “Online STI Testing” [53], and “Take a free test” [54]. These HIV and STI prediction tools provide only subjective terms such as “high” risk or “You are advised to take an HIV/STI test.” Our risk prediction tool could quantify the risk of HIV and STIs. In addition, our artificial intelligence (AI)–based risk prediction tool can simultaneously provide risk scores for HIV and 3 common STIs (gonorrhea, chlamydia, and syphilis) for men and women aged 18 years and older.

### Implications

Our web-based HIV and STI risk prediction tool can be used as a screening tool to potentially increase HIV and STI testing and encourage access to testing and health care (Figure S8 in Multimedia Appendix 1). The tool could be used on clinic websites so the public could assess their risk and then decide if they would attend a clinic for testing. It may also be used within a clinic to identify and triage those at higher risk of HIV and STIs if the demand in the clinic is too great to see everyone who attended. However, an AI-based risk prediction tool cannot replace formal HIV and STI testing and treatment in clinical settings, but it would allow individuals to understand their own risks and increase testing uptake. Our tool could increase risk perception and concern about infection, thus increasing HIV and STI testing. A study in the British population showed that increased risk perceptions are associated with greater STI health care use [12]. Further external validation of our AI-based risk prediction tool in other countries or regions, such as low- and middle-income countries, may provide an opportunity to reduce the cost of HIV and STI screening by better focusing testing on those at highest risk [55].

There are many possible ways that our web-based risk prediction tool could be potentially used, including as part of a behavioral intervention to control HIV and STIs or to help clinicians or public health workers identify high-risk individuals for risk management or further interventions. An example of this exists in adolescent health risk behaviors. Researchers used an individual’s risk behavior scores and personalized feedback as part of an intervention for health behaviors, including nutritional behaviors, physical activity, and sleep [56]. In this randomized clinical trial, the youths in the intervention group significantly reduced their risk behavior scores at 3 months compared with the control group [56]. Our web-based risk prediction tool could serve as a behavioral intervention tool in the same way.

Future work will investigate the effectiveness of this web-based HIV and STI risk prediction tool for behavioral change (ie, uptake of PrEP or condom promotion) and STI service utilization behaviors (timely clinic attendance and HIV and STI testing uptake) after receiving risk prediction results and testing recommendations. Implementing this web-based HIV and STI prediction tool may encourage individuals with STI symptoms or those at high risk without symptoms to attend health services for timely testing and regular testing. Since February 2009, the MSHC has offered MSM regular SMS reminders for STI screening [57]. For example, providing an estimated risk of HIV and STIs and risk reduction advice (ie, uptake of PrEP or condom promotion) among high-risk populations (eg, MSM) in an SMS reminder message may encourage testing and behavioral changes.

### Limitations

This study has some limitations. First, the predictive factors depend on self-reported information from the CASI system, which is subject to the participants' recall, nonresponse, and social desirability bias. For example, MSM who declined to report the number of male partners were at a higher risk of chlamydia [58]. There has been substantial work undertaken on the CASI system's validity and accuracy [59]. Second, machine learning models may suffer from overfitting. We used repeated CV to tackle the overfitting problem. We also used ensemble learning methods to enhance the model's generalizability. Third, the generalizability of our models to those not attending the clinic or to other countries or regions is limited because it was derived from a single sexual health service. Thus, if it is used in other countries and regions, further validation is required. Finally, the risks of HIV have changed rapidly over this time by introducing PrEP, so future models will need to include this question, given how the potency of this single preventive strategy.

### Conclusions

This is the first web-based risk assessment tool using machine learning algorithms and self-reported data to identify HIV, syphilis, gonorrhea, and chlamydia in men and women. Our online risk prediction tool could accurately predict the risk of HIV and STIs in clinic attendees with a simple self-administered questionnaire. Our risk prediction tool could be part of clinic websites or digital health platforms. The public can use this risk prediction tool to assess their HIV and STI risk to inform testing. Clinicians or public health workers can use this risk prediction tool to identify high-risk individuals for further interventions.

## Appendix

Multimedia Appendix 1 Supplementary tables and figures.

## Abbreviations

AI: artificial intelligence
AUC: area under the curve
CASI: computer-assisted self-interview system
CV: cross-validation
DL: deep learning
EHR: electronic health records
ENR: elastic net regression
GBM: gradient boosting machine
LASSO: least absolute shrinkage and selection operator
MSHC: Melbourne Sexual Health Centre
MSM: men who have sex with men
NAAT: nucleic amplification tests
NB: Naive Bayes
PrEP: pre-exposure prophylaxis
RF: random forest
RR: ridge regression
STI: sexually transmitted infection
WHO: World Health Organization
