# Supplementary material for: Web-Based Risk Prediction Tool for an Individual's Risk of HIV and Sexually Transmitted Infections Using Machine Learning Algorithms: Development and External Validation Study
Source: J Med Internet Res. 2022 Aug 25;24(8):e37850. doi: 10.2196/37850 (PMC9459839; doi:10.2196/37850)

**Characteristics of training, testing, and external validation data**

**Characteristics of training and testing data**

Table S1. Characteristics of clinic consultations in the training and testing data from March 2, 2015 to December 31, 2018

| **Variables** | **HIV**  **(n=88,642 consultations)** | **Syphilis**  **(n=92,291 consultations)** | **Gonorrhoea**  **(n=97,473 consultations)** | **Chlamydia**  **(n=115,845 consultations)** |
| --- | --- | --- | --- | --- |
| **Pre-exposure prophylaxis (PrEP) use** |  |  |  |  |
| No | 86560 (97.7%) | 89868 (97.4%) | 94475 (96.9%) | 112905 (97.5%) |
| Yes | 2082 (2.3%) | 2423 (2.6%) | 2998 (3.1%) | 2940 (2.5%) |
| ***Sexual Practices*** |  |  |  |  |
| **Men who have sex with men** |  |  |  |  |
| Not applicable (female) | 26651 (30.1%) | 27134 (29.4%) | 31282 (32.1%) | 38548 (33.3%) |
| No | 16508 (18.6%) | 17089 (18.5%) | 15245 (15.6%) | 26975 (23.3%) |
| Yes | 45483 (51.3%) | 48068 (52.1%) | 50946 (52.3%) | 50322 (43.4%) |
| **Have sex with a male in the last 12 months** |  |  |  |  |
| No | 21412 (24.2%) | 22080 (23.9%) | 20421 (21.0%) | 32561 (28.1%) |
| Yes | 64579 (72.9%) | 67273 (72.9%) | 73887 (75.8%) | 80024 (69.1%) |
| Unknown/ Missing | 2651 (3.0%) | 2938 (3.2%) | 3165 (3.2%) | 3260 (2.8%) |
| **Condoms use with male partners in the last 12 months** |  |  |  |  |
| Always | 18330 (20.7%) | 18825 (20.4%) | 19895 (20.4%) | 20490 (17.7%) |
| Never | 5342 (6.0%) | 5638 (6.1%) | 6518 (6.7%) | 7128 (6.2%) |
| Not applicable (no vag/anal sex) | 1318 (1.5%) | 1378 (1.5%) | 1479 (1.5%) | 1488 (1.3%) |
| Unknown or Declined to answer | 3164 (3.6%) | 3540 (3.8%) | 3826 (3.9%) | 4009 (3.5%) |
| Usually or Sometimes | 37819 (42.7%) | 39485 (42.8%) | 43943 (45.1%) | 48764 (42.1%) |
| Declined to answer /Missing | 22669 (25.6%) | 23425 (25.4%) | 21812 (22.4%) | 33966 (29.3%) |
| **Have sex with a female in the last 12 months** |  |  |  |  |
| No | 61291 (69.1%) | 63919 (69.3%) | 70036 (71.9%) | 75869 (65.5%) |
| Yes | 24825 (28.0%) | 25572 (27.7%) | 24358 (25.0%) | 36827 (31.8%) |
| Unknown or Declined to answer /Missing | 2526 (2.8%) | 2800 (3.0%) | 3079 (3.2%) | 3149 (2.7%) |
| **Condoms use with female partners in the last 12 months** |  |  |  |  |
| Always | 3983 (4.5%) | 4120 (4.5%) | 3642 (3.7%) | 5328 (4.6%) |
| Never | 2722 (3.1%) | 2860 (3.1%) | 2811 (2.9%) | 4125 (3.6%) |
| Not applicable (no vag/anal sex) | 2159 (2.4%) | 2247 (2.4%) | 2555 (2.6%) | 2727 (2.4%) |
| Unknown or Declined to answer | 1670 (1.9%) | 1953 (2.1%) | 2072 (2.1%) | 2251 (1.9%) |
| Usually (>50%) or Sometimes | 14437 (16.3%) | 14843 (16.1%) | 13766 (14.1%) | 22544 (19.5%) |
| Declined to answer/ Missing | 63671 (71.8%) | 66268 (71.8%) | 72627 (74.5%) | 78870 (68.1%) |
| **Sex oversea (outside Australia or New Zealand) in the last 12 months** |  |  |  |  |
| No | 49436 (55.8%) | 51496 (55.8%) | 55300 (56.7%) | 64794 (55.9%) |
| Yes | 30968 (34.9%) | 31962 (34.6%) | 32689 (33.5%) | 40917 (35.3%) |
| Missing | 8238 (9.3%) | 8833 (9.6%) | 9484 (9.7%) | 10134 (8.7%) |
| **Current sex worker** |  |  |  |  |
| No | 80848 (91.2%) | 84466 (91.5%) | 89302 (91.6%) | 107698 (93.0%) |
| Yes | 7794 (8.8%) | 7825 (8.5%) | 8171 (8.4%) | 8147 (7.0%) |
| **Number of casual male sexual partners in the last 12 months** |  |  |  |  |
| Median (IQR) | 5.0 (2.0- 10.0) | 5.0(2.0-10) | 5.0(2.0-10.0) | 5.0(2.0-9.0) |
| Not applicable (no male sexual partners) / Unknown or Declined to answer | 25783 (29.1%) | 26887 (29.1%) | 25564 (26.2%) | 37912 (32.7%) |
| **Number of casual female sexual partners in the last 12 months** |  |  |  |  |
| Median (IQR) | 3.0(2.0-6.0) | 3.0(2.0-6.0) | 3.0(2.0-6.0) | 3.0(2.0-6.0) |
| Not applicable (no male sexual partners) / Unknown or Declined to answer | 64460 (72.7%) | 67376 (73.0%) | 73730 (75.6%) | 79831 (68.9%) |
| ***Infection contact history*** |  |  |  |  |
| **Contact with a gonorrhoea case** |  |  |  |  |
| No/Missing | 86552 (97.6%) | 89992 (97.5%) | 94662 (97.1%) | 113074 (97.6%) |
| Yes | 2090 (2.4%) | 2299 (2.5%) | 2811 (2.9%) | 2771 (2.4%) |
| **Contact with a chlamydia case** |  |  |  |  |
| No/Missing | 86138 (97.2%) | 89628 (97.1%) | 94057 (96.5%) | 111353 (96.1%) |
| Yes | 2504 (2.8%) | 2663 (2.9%) | 3416 (3.5%) | 4492 (3.9%) |
| **Contact with a syphilis case** |  |  |  |  |
| No/Missing | 87813 (99.1%) | 91287 (98.9%) | 96502 (99.0%) | 114867 (99.2%) |
| Yes | 829 (0.9%) | 1004 (1.1%) | 971 (1.0%) | 978 (0.8%) |
| ***Infection history*** |  |  |  |  |
| **Past genital Warts infection** |  |  |  |  |
| No | 26082 (29.4%) | 27985 (30.3%) | 31506 (32.3%) | 34961 (30.2%) |
| Yes | 5588 (6.3%) | 5980 (6.5%) | 6469 (6.6%) | 7586 (6.5%) |
| Unsure/Unknown /Missing | 56972 (64.3%) | 58326 (63.2%) | 59498 (61.0%) | 73298 (63.3%) |
| **Past chlamydia infection** |  |  |  |  |
| No | 13489 (15.2%) | 14515 (15.7%) | 15538 (15.9%) | 16920 (14.6%) |
| Yes | 18181 (20.5%) | 19450 (21.1%) | 22437 (23.0%) | 25627 (22.1%) |
| Missing | 56972 (64.3%) | 58326 (63.2%) | 59498 (61.0%) | 73298 (63.3%) |
| **Past gonorrhoea infection** |  |  |  |  |
| No | 19111 (21.6%) | 20250 (21.9%) | 22495 (23.1%) | 27349 (23.6%) |
| Yes | 12559 (14.2%) | 13715 (14.9%) | 15480 (15.9%) | 15198 (13.1%) |
| Unsure/Unknown /Missing | 56972 (64.3%) | 58326 (63.2%) | 59498 (61.0%) | 73298 (63.3%) |
| **Past non-specific urethritis (NSU) infection** |  |  |  |  |
| No | 24050 (27.1%) | 26048 (28.2%) | 28180 (28.9%) | 30627 (26.4%) |
| Yes | 857 (1.0%) | 952 (1.0%) | 1100 (1.1%) | 1151 (1.0%) |
| Missing | 63735 (71.9%) | 65291 (70.7%) | 68193 (70.0%) | 84067 (72.6%) |
| **Past syphilis infection** |  |  |  |  |
| No | 27119 (30.6%) | 28478 (30.9%) | 32212 (33.0%) | 36846 (31.8%) |
| Yes | 4551 (5.1%) | 5487 (5.9%) | 5763 (5.9%) | 5701 (4.9%) |
| Unsure/Unknown /Missing | 56972 (64.3%) | 58326 (63.2%) | 59498 (61.0%) | 73298 (63.3%) |
| **Past genital Herpes infection** |  |  |  |  |
| No | 29766 (33.6%) | 31917 (34.6%) | 35713 (36.6%) | 39856 (34.4%) |
| Yes | 3548 (4.0%) | 3866 (4.2%) | 4247 (4.4%) | 4819 (4.2%) |
| Unsure/Unknown /Missing | 55328 (62.4%) | 56508 (61.2%) | 57513 (59.0%) | 71170 (61.4%) |
| **HIV infection** | **-** |  |  |  |
| HIV-negative | - | 32667 (35.4%) | 36238 (37.2%) | 40899 (35.3%) |
| Living with HIV | - | 1298 (1.4%) | 1737 (1.8%) | 1648 (1.4%) |
| Missing/Unsure/Unknown | - | 58326 (63.2%) | 59498 (61.0%) | 73298 (63.3%) |
| **Past Hepatitis B infection** |  |  |  |  |
| No | 31314 (35.3%) | 33539 (36.3%) | 37533 (38.5%) | 42083 (36.3%) |
| Yes | 356 (0.4%) | 426 (0.5%) | 442 (0.5%) | 464 (0.4%) |
| Unsure/Unknown /Missing | 56972 (64.3%) | 58326 (63.2%) | 59498 (61.0%) | 73298 (63.3%) |
| **Past Hepatitis C infection** |  |  |  |  |
| No | 31482 (35.5%) | 33704 (36.5%) | 37683 (38.7%) | 42246 (36.5%) |
| Yes | 188 (0.2%) | 261 (0.3%) | 292 (0.3%) | 301 (0.3%) |
| Unsure/Unknown /Missing | 56972 (64.3%) | 58326 (63.2%) | 59498 (61.0%) | 73298 (63.3%) |
| **Other past STI infections** |  |  |  |  |
| No | 31482 (35.5%) | 33704 (36.5%) | 37683 (38.7%) | 42246 (36.5%) |
| Yes | 188 (0.2%) | 261 (0.3%) | 292 (0.3%) | 301 (0.3%) |
| Unsure/Unknown /Missing | 56972 (64.3%) | 58326 (63.2%) | 59498 (61.0%) | 73298 (63.3%) |
| ***Drug injection*** |  |  |  |  |
| Less than 12 months ago | 372 (0.4%) | 419 (0.5%) | 455 (0.5%) | 486 (0.4%) |
| Less than 3 months ago | 1008 (1.1%) | 1154 (1.3%) | 1243 (1.3%) | 1284 (1.1%) |
| More than 12 months ago | 976 (1.1%) | 1061 (1.1%) | 1089 (1.1%) | 1217 (1.1%) |
| Never injected | 83634 (94.4%) | 86658 (93.9%) | 91452 (93.8%) | 109480 (94.5%) |
| Declined to answer/ Unknown/ Missing | 2652 (3.0%) | 2999 (3.2%) | 3234 (3.3%) | 3378 (2.9%) |

^a^ IQR: interquartile range

^b^ STI: sexually transmitted infections

**Characteristics of external validation analysis data**

Our external validation data including [57/28875= 0.20% external validation data in 2019, 27 /18052= 0.15% external validation data in 2020 and January, 2021] HIV positive infections, [586/30302 =1.93% external validation data in 2019, 507/19150= 2.65% external validation data in 2020 and January, 2021] syphilis positive infections, [2303 /36805 = 6.26% external validation data in 2019, 1565/22886= 6.84% external validation data in 2020 and January, 2021] gonorrhoea positive infections, and [3231/36393= 8.88% external validation data in 2019,1498 /22615=6.62 % external validation data in 2020 and January, 2021] chlamydia positive infections.

Table S2. Characteristics of HIV on external validation data (2019) and external validation data (2020 - 2021)

| **Variables** | External validation data  (2019) | External validation data  (2020 - 2021) |
| --- | --- | --- |
| **Gender** |  |  |
| Female | 9367 (32.4%) | 5114 (28.3%) |
| Male | 19508 (67.6%) | 12938 (71.7%) |
| **Men who have sex with men (men only)** |  |  |
| No | 5906 (30.3%) | 3473 (26.8%) |
| Yes | 13602 (69.7%) | 9465 (73.2%) |
| **Country of birth** |  |  |
| Australia | 11407 (39.5%) | 7140 (39.6%) |
| Oversea | 16317 (56.5%) | 9294 (51.5%) |
| Missing | 1151 (4.0%) | 1618 (9.0%) |
| **Have sex with a male in the last 12 months** |  |  |
| No | 7303 (25.3%) | 4151 (23.0%) |
| Yes | 20776 (72.0%) | 13214 (73.2%) |
| Missing | 796 (2.8%) | 687 (3.8%) |
| **Condoms use with male partners in the last 12 months** |  |  |
| Always | 4724 (16.4%) | 2606 (14.4%) |
| Never | 2096 (7.3%) | 1562 (8.7%) |
| Not applicable (no vag/anal sex) | 510 (1.8%) | 299 (1.7%) |
| Unknown or Declined to answer | 1011 (3.5%) | 572 (3.2%) |
| Usually (>50%) or Sometimes | 12933 (44.8%) | 8347 (46.2%) |
| Missing | 7601 (26.3%) | 4666 (25.8%) |
| **Have sex with a female in the last 12 months** |  |  |
| No | 19271 (66.7%) | 12183 (67.5%) |
| Yes | 8798 (30.5%) | 5147 (28.5%) |
| Missing | 806 (2.8%) | 722 (4.0%) |
| **Condoms use with female partners in the last 12 months** |  |  |
| Always | 1380 (4.8%) | 853 (4.7%) |
| Never | 986 (3.4%) | 584 (3.2%) |
| Not applicable (no vag/anal sex) | 8620 (29.9%) | 6491 (36.0%) |
| Unknown or Declined to answer | 610 (2.1%) | 347 (1.9%) |
| Usually (>50%) or Sometimes | 5018 (17.4%) | 2954 (16.4%) |
| Missing | 12261 (42.5%) | 6823 (37.8%) |
| ***Drug injection*** |  |  |
| Less than 12 months ago | 95 (0.3%) | 76 (0.4%) |
| Less than 3 months ago | 260 (0.9%) | 220 (1.2%) |
| More than 12 months ago | 237 (0.8%) | 142 (0.8%) |
| Never injected | 27373 (94.8%) | 16997 (94.2%) |
| Missing | 910 (3.2%) | 617 (3.4%) |
| **Pre-exposure prophylaxis (PrEP) use** |  |  |
| No | 27106 (93.9%) | 16071 (89.0%) |
| Yes | 1769 (6.1%) | 1981 (11.0%) |
| **Sex oversea (outside Australia or New Zealand) in the last 12 months** |  |  |
| No | 15161 (52.5%) | 11358 (62.9%) |
| Yes | 11088 (38.4%) | 4812 (26.7%) |
| Missing | 2626 (9.1%) | 1882 (10.4%) |
| **Current sex worker** |  |  |
| No | 26528 (91.9%) | 17353 (96.1%) |
| Yes | 2347 (8.1%) | 699 (3.9%) |
| **Contact with a gonorrhoea case** |  |  |
| No/Missing | 28359 (98.2%) | 17493 (96.9%) |
| Yes | 516 (1.8%) | 559 (3.1%) |
| **Contact with a chlamydia case** |  |  |
| No/Missing | 28006 (97.0%) | 17083 (94.6%) |
| Yes | 869 (3.0%) | 969 (5.4%) |
| **Contact with a syphilis case** |  |  |
| No/Missing | 28605 (99.1%) | 17743 (98.3%) |
| Yes | 270 (0.9%) | 309 (1.7%) |
| **STI symptom** |  |  |
| No | 18042 (62.5%) | 4435 (24.6%) |
| Yes | 8072 (28.0%) | 6515 (36.1%) |
| Missing | 2761 (9.6%) | 7102 (39.3%) |
| **Past genital warts infection** |  |  |
| No | 8527 (29.5%) | 6062 (33.6%) |
| Yes | 1596 (5.5%) | 1094 (6.1%) |
| Missing | 18752 (64.9%) | 10896 (60.4%) |
| **Past chlamydia infection** |  |  |
| No | 3920 (13.6%) | 2690 (14.9%) |
| Yes | 6203 (21.5%) | 4466 (24.7%) |
| Missing | 18752 (64.9%) | 10896 (60.4%) |
| **Past gonorrhoea infection** |  |  |
| No | 6028 (20.9%) | 3910 (21.7%) |
| Yes | 4095 (14.2%) | 3246 (18.0%) |
| Missing | 18752 (64.9%) | 10896 (60.4%) |
| **Past non-specific urethritis infection** |  |  |
| No | 7358 (25.5%) | 5389 (29.9%) |
| Yes | 208 (0.7%) | 173 (1.0%) |
| Missing | 21309 (73.8%) | 12490 (69.2%) |
| **Past syphilis infection** |  |  |
| No | 8649 (30.0%) | 5898 (32.7%) |
| Yes | 1474 (5.1%) | 1258 (7.0%) |
| Missing | 18752 (64.9%) | 10896 (60.4%) |
| **Past genital Herpes** |  |  |
| No | 9539 (33.0%) | 6679 (37.0%) |
| Yes | 1134 (3.9%) | 795 (4.4%) |
| Missing | 18202 (63.0%) | 10578 (58.6%) |
| **Past Hepatitis B infection** |  |  |
| No | 10017 (34.7%) | 7087 (39.3%) |
| Yes | 106 (0.4%) | 69 (0.4%) |
| Missing | 18752 (64.9%) | 10896 (60.4%) |
| **Past Hepatitis C infection** |  |  |
| No | 10068 (34.9%) | 7119 (39.4%) |
| Yes | 55 (0.2%) | 37 (0.2%) |
| Missing | 18752 (64.9%) | 10896 (60.4%) |
| **Other past infections** |  |  |
| No | 10068 (34.9%) | 7119 (39.4%) |
| Yes | 55 (0.2%) | 37 (0.2%) |
| Missing | 18752 (64.9%) | 10896 (60.4%) |
| **Age at consultation** |  |  |
| Median (IQR) | 28.0(24.0-34.0) | 29.0(25.0-35.0) |
| **Number of casual male sexual partners in the last 12 months** |  |  |
| Median (IQR) | 4.0(2.0-8.0) | 4.0(2.0-9.0) |
| Missing (Not applicable (no male sexual partners) / Unknown or Declined to answer) | 8730 (30.2%) | 5195 (28.8%) |
| **Number of casual female sexual partners in the last 12 months** |  |  |
| Median (IQR) | 3.0(2.0-6.0) | 3.0(2.0-5.0) |
| Missing (Not applicable (no male sexual partners) / Unknown or Declined to answer) | 20384 (70.6%) | 13086 (72.5%) |

^a^ IQR: interquartile range

^b^ STI: sexually transmitted infections

Table S3. Characteristics of syphilis on external validation data (2019) and external validation data (2020 - 2021)

| **Variables** | External validation data  (2019) | External validation data  (2020 - 2021) |
| --- | --- | --- |
| **Gender** |  |  |
| Female | 9629 (31.8%) | 5286 (27.6%) |
| Male | 20673 (68.2%) | 13864 (72.4%) |
| **Men who have sex with men (men only)** |  |  |
| No | 6152 (29.8%) | 3632 (26.2%) |
| Yes | 14521 (70.2%) | 10232 (73.8%) |
| **Country of birth** |  |  |
| Australia | 12077 (39.9%) | 7695 (40.2%) |
| Oversea | 17030 (56.2%) | 9790 (51.1%) |
| Missing | 1195 (3.9%) | 1665 (8.7%) |
| **Have sex with a male in the last 12 months** |  |  |
| No | 7587 (25.0%) | 4335 (22.6%) |
| Yes | 21808 (72.0%) | 14036 (73.3%) |
| Missing | 907 (3.0%) | 779 (4.1%) |
| **Condoms use with male partners in the last 12 months** |  |  |
| Always | 4866 (16.1%) | 2686 (14.0%) |
| Never | 2249 (7.4%) | 1704 (8.9%) |
| Not applicable (no vag/anal sex) | 528 (1.7%) | 319 (1.7%) |
| Unknown or Declined to answer | 1135 (3.7%) | 676 (3.5%) |
| Usually (>50%) or Sometimes | 13600 (44.9%) | 8883 (46.4%) |
| Missing | 7924 (26.2%) | 4882 (25.5%) |
| **Have sex with a female in the last 12 months** |  |  |
| No | 20270 (66.9%) | 12991 (67.8%) |
| Yes | 9114 (30.1%) | 5350 (27.9%) |
| Missing | 918 (3.0%) | 809 (4.2%) |
| **Condoms use with female partners in the last 12 months** |  |  |
| Always | 1432 (4.7%) | 888 (4.6%) |
| Never | 1052 (3.5%) | 630 (3.3%) |
| Not applicable (no vag/anal sex) | 9050 (29.9%) | 6913 (36.1%) |
| Unknown or Declined to answer | 706 (2.3%) | 428 (2.2%) |
| Usually (>50%) or Sometimes | 5187 (17.1%) | 3055 (16.0%) |
| Missing | 12875 (42.5%) | 7236 (37.8%) |
| ***Drug injection*** |  |  |
| Less than 12 months ago | 110 (0.4%) | 87 (0.5%) |
| Less than 3 months ago | 313 (1.0%) | 257 (1.3%) |
| More than 12 months ago | 256 (0.8%) | 167 (0.9%) |
| Never injected | 28559 (94.2%) | 17899 (93.5%) |
| Missing | 1064 (3.5%) | 740 (3.9%) |
| **Pre-exposure prophylaxis (PrEP) use** |  |  |
| No | 28404 (93.7%) | 17072 (89.1%) |
| Yes | 1898 (6.3%) | 2078 (10.9%) |
| **Sex oversea (outside Australia or New Zealand) in the last 12 months** |  |  |
| No | 15904 (52.5%) | 12026 (62.8%) |
| Yes | 11536 (38.1%) | 5036 (26.3%) |
| Missing | 2862 (9.4%) | 2088 (10.9%) |
| **Current sex worker** |  |  |
| No | 27939 (92.2%) | 18447 (96.3%) |
| Yes | 2363 (7.8%) | 703 (3.7%) |
| **Contact with a gonorrhoea case** |  |  |
| No/Missing | 29727 (98.1%) | 18507 (96.6%) |
| Yes | 575 (1.9%) | 643 (3.4%) |
| **Contact with a chlamydia case** |  |  |
| No/Missing | 29378 (97.0%) | 18116 (94.6%) |
| Yes | 924 (3.0%) | 1034 (5.4%) |
| **Contact with a syphilis case** |  |  |
| No/Missing | 29966 (98.9%) | 18782 (98.1%) |
| Yes | 336 (1.1%) | 368 (1.9%) |
| **STI symptom** |  |  |
| No | 18548 (61.2%) | 4765 (24.9%) |
| Yes | 8882 (29.3%) | 7140 (37.3%) |
| Missing | 2872 (9.5%) | 7245 (37.8%) |
| **Past genital warts infection** |  |  |
| No | 9197 (30.4%) | 6651 (34.7%) |
| Yes | 1742 (5.7%) | 1219 (6.4%) |
| Missing | 19363 (63.9%) | 11280 (58.9%) |
| **Past chlamydia infection** |  |  |
| No | 4289 (14.2%) | 2991 (15.6%) |
| Yes | 6650 (21.9%) | 4879 (25.5%) |
| Missing | 19363 (63.9%) | 11280 (58.9%) |
| **Past gonorrhoea infection** |  |  |
| No | 6416 (21.2%) | 4257 (22.2%) |
| Yes | 4523 (14.9%) | 3613 (18.9%) |
| Missing | 19363 (63.9%) | 11280 (58.9%) |
| **Past non-specific urethritis infection** |  |  |
| No | 8058 (26.6%) | 5999 (31.3%) |
| Yes | 234 (0.8%) | 194 (1.0%) |
| Missing | 22010 (72.6%) | 12957 (67.7%) |
| **Past syphilis infection** |  |  |
| No | 9137 (30.2%) | 6320 (33.0%) |
| Yes | 1802 (5.9%) | 1550 (8.1%) |
| Missing | 19363 (63.9%) | 11280 (58.9%) |
| **Past genital Herpes infection** |  |  |
| No | 10280 (33.9%) | 7320 (38.2%) |
| Yes | 1263 (4.2%) | 893 (4.7%) |
| Missing | 18759 (61.9%) | 10937 (57.1%) |
| **HIV infection** |  |  |
| HIV-negative | 10506 (34.7%) | 7514 (39.2%) |
| Living with HIV | 433 (1.4%) | 356 (1.9%) |
| Missing | 19363 (63.9%) | 11280 (58.9%) |
| **Past Hepatitis B infection** |  |  |
| No | 10822 (35.7%) | 7790 (40.7%) |
| Yes | 117 (0.4%) | 80 (0.4%) |
| Missing | 19363 (63.9%) | 11280 (58.9%) |
| **Past Hepatitis C infection** |  |  |
| No | 10860 (35.8%) | 7819 (40.8%) |
| Yes | 79 (0.3%) | 51 (0.3%) |
| Missing | 19363 (63.9%) | 11280 (58.9%) |
| **Other past STI infections** |  |  |
| No | 10860 (35.8%) | 7819 (40.8%) |
| Yes | 79 (0.3%) | 51 (0.3%) |
| Missing | 19363 (63.9%) | 11280 (58.9%) |
| **Age at consultation** |  |  |
| Median (IQR) | 28.0(24.0-34) | 29.0(25.0-35.0) |
| **Number of casual male sexual partners in the last 12 months** |  |  |
| Median (IQR) | 5.0(2.0-9.0) | 4.0(2.0-9.0) |
| Missing (Not applicable (no male sexual partners) / Unknown or Declined to answer) | 9177 (30.3%) | 5512 (28.8%) |
| **Number of casual female sexual partners in the last 12 months** |  |  |
| Median (IQR ) | 3.0(2.0-6.0) | 3.0(2.0-5.0) |
| Missing (Not applicable (no male sexual partners) / Unknown or Declined to answer ) | 21504 (71.0%) | 13988 (73.0%) |

^a^ IQR: interquartile range

^b^ STI: sexually transmitted infections

Table S4. Characteristics of gonorrhoea on external validation data (2019) and external validation data (2020 - 2021)

| **Variables** | External validation data  (2019) | External validation data  (2020 - 2021) |
| --- | --- | --- |
| **Gender** |  |  |
| Female | 12722 (34.6%) | 7046 (30.8%) |
| Male | 24083 (65.4%) | 15840 (69.2%) |
| **Men who have sex with men (men only)** |  |  |
| No | 8321 (34.6%) | 4804 (30.3%) |
| Yes | 15762 (65.4%) | 11036 (69.7%) |
| **Country of birth** |  |  |
| Australia | 14670 (39.9%) | 9260 (40.5%) |
| Oversea | 20708 (56.3%) | 11665 (51.0%) |
| Missing | 1427 (3.9%) | 1961 (8.6%) |
| **Have sex with a male in the last 12 months** |  |  |
| No | 9912 (26.9%) | 5573 (24.4%) |
| Yes | 25854 (70.2%) | 16423 (71.8%) |
| Missing | 1039 (2.8%) | 890 (3.9%) |
| **Condoms use with male partners in the last 12 months** |  |  |
| Always | 5395 (14.7%) | 3009 (13.1%) |
| Never | 2711 (7.4%) | 2052 (9.0%) |
| Not applicable (no vag/anal sex) | 605 (1.6%) | 372 (1.6%) |
| Unknown or Declined to answer | 1335 (3.6%) | 787 (3.4%) |
| Usually (>50%) or Sometimes | 16524 (44.9%) | 10530 (46.0%) |
| Missing | 10235 (27.8%) | 6136 (26.8%) |
| **Have sex with a female in the last 12 months** |  |  |
| No | 24071 (65.4%) | 15200 (66.4%) |
| Yes | 11685 (31.7%) | 6776 (29.6%) |
| Missing | 1049 (2.9%) | 910 (4.0%) |
| **Condoms use with female partners in the last 12 months** |  |  |
| Always | 1708 (4.6%) | 1043 (4.6%) |
| Never | 1347 (3.7%) | 801 (3.5%) |
| Not applicable (no vag/anal sex) | 9700 (26.4%) | 7453 (32.6%) |
| Unknown or Declined to answer | 864 (2.3%) | 500 (2.2%) |
| Usually (>50%)or Sometimes | 6833 (18.6%) | 3924 (17.1%) |
| Missing | 16353 (44.4%) | 9165 (40.0%) |
| ***Drug injection*** |  |  |
| Less than 12 months ago | 122 (0.3%) | 102 (0.4%) |
| Less than 3 months ago | 362 (1.0%) | 279 (1.2%) |
| More than 12 months ago | 305 (0.8%) | 180 (0.8%) |
| Never injected | 34818 (94.6%) | 21466 (93.8%) |
| Missing | 1198 (3.3%) | 859 (3.8%) |
| **Pre-exposure prophylaxis (PrEP) use** |  |  |
| No | 34562 (93.9%) | 20589 (90.0%) |
| Yes | 2243 (6.1%) | 2297 (10.0%) |
| **Sex oversea (outside Australia or New Zealand) in the last 12 months** |  |  |
| No | 19327 (52.5%) | 14431 (63.1%) |
| Yes | 14188 (38.5%) | 6107 (26.7%) |
| Missing | 3290 (8.9%) | 2348 (10.3%) |
| **Current sex worker** |  |  |
| No | 34310 (93.2%) | 22137 (96.7%) |
| Yes | 2495 (6.8%) | 749 (3.3%) |
| **Contact with a gonorrhoea case** |  |  |
| No/Missing | 36058 (98.0%) | 22066 (96.4%) |
| Yes | 747 (2.0%) | 820 (3.6%) |
| **Contact with a chlamydia case** |  |  |
| No/Missing | 35380 (96.1%) | 21487 (93.9%) |
| Yes | 1425 (3.9%) | 1399 (6.1%) |
| **Contact with a syphilis case** |  |  |
| No/Missing | 36491 (99.1%) | 22526 (98.4%) |
| Yes | 314 (0.9%) | 360 (1.6%) |
| **STI symptom** |  |  |
| No | 21736 (59.1%) | 5598 (24.5%) |
| Yes | 11959 (32.5%) | 9479 (41.4%) |
| Missing | 3110 (8.4%) | 7809 (34.1%) |
| **Past genital warts infection** |  |  |
| No | 11430 (31.1%) | 8128 (35.5%) |
| Yes | 2146 (5.8%) | 1462 (6.4%) |
| Missing | 23229 (63.1%) | 13296 (58.1%) |
| **Past chlamydia infection** |  |  |
| No | 5003 (13.6%) | 3479 (15.2%) |
| Yes | 8573 (23.3%) | 6111 (26.7%) |
| Missing | 23229 (63.1%) | 13296 (58.1%) |
| **Past gonorrhoea infection** |  |  |
| No | 8304 (22.6%) | 5419 (23.7%) |
| Yes | 5272 (14.3%) | 4171 (18.2%) |
| Missing | 23229 (63.1%) | 13296 (58.1%) |
| **Past non-specific urethritis infection** |  |  |
| No | 9532 (25.9%) | 6981 (30.5%) |
| Yes | 288 (0.8%) | 229 (1.0%) |
| Missing | 26985 (73.3%) | 15676 (68.5%) |
| **Past syphilis infection** |  |  |
| No | 11638 (31.6%) | 7963 (34.8%) |
| Yes | 1938 (5.3%) | 1627 (7.1%) |
| Missing | 23229 (63.1%) | 13296 (58.1%) |
| **Past genital herpes infection** |  |  |
| No | 12755 (34.7%) | 8937 (39.1%) |
| Yes | 1511 (4.1%) | 1071 (4.7%) |
| Missing | 22539 (61.2%) | 12878 (56.3%) |
| **HIV infection** |  |  |
| HIV-negative | 13005 (35.3%) | 9122 (39.9%) |
| Living with HIV | 571 (1.6%) | 468 (2.0%) |
| Missing | 23229 (63.1%) | 13296 (58.1%) |
| **Past hepatitis B infection** |  |  |
| No | 13442 (36.5%) | 9502 (41.5%) |
| Yes | 134 (0.4%) | 88 (0.4%) |
| Missing | 23229 (63.1%) | 13296 (58.1%) |
| **Past hepatitis C infection** |  |  |
| No | 13494 (36.7%) | 9537 (41.7%) |
| Yes | 82 (0.2%) | 53 (0.2%) |
| Missing | 23229 (63.1%) | 13296 (58.1%) |
| **Other past STI infections** |  |  |
| No | 13494 (36.7%) | 9537 (41.7%) |
| Yes | 82 (0.2%) | 53 (0.2%) |
| Missing | 23229 (63.1%) | 13296 (58.1%) |
| **Age at consultation** |  |  |
| Median (IQR) | 28.0(24.0-34.0) | 29.0(25.0-34.0) |
| **Number of casual male sexual partners in the last 12 months** |  |  |
| Median (IQR) | 4.0(2.0-8.0) | 4.0(2.0-8.0) |
| Missing (Not applicable (no male sexual partners) / Unknown or Declined to answer) | 11700 (31.8%) | 6917 (30.2%) |
| **Number of casual female sexual partners in the last 12 months** |  |  |
| Median (IQR) | 3.0(2.0-6.0) | 3.0(2.0-6.0) |
| Missing (Not applicable (no male sexual partners) / Unknown or Declined to answer) | 25509 (69.3%) | 16350 (71.4%) |

^a^ IQR: interquartile range

^b^ STI: sexually transmitted infections

Table S5. Characteristics of chlamydia on external validation data (2019) and external validation data (2020 - 2021)

| **Variables** | External validation data  (2019) | External validation data  (2020 - 2021) |
| --- | --- | --- |
| **Gender** |  |  |
| Female | 12680 (34.8%) | 7039 (31.1%) |
| Male | 23713 (65.2%) | 15576 (68.9%) |
| **Men who have sex with men (men only)** |  |  |
| No | 8320 (35.1%) | 4798 (30.8%) |
| Yes | 15393 (64.9%) | 10778 (69.2%) |
| **Country of birth** |  |  |
| Australia | 14492 (39.8%) | 9151 (40.5%) |
| Oversea | 20487 (56.3%) | 11509 (50.9%) |
| Missing | 1414 (3.9%) | 1955 (8.6%) |
| **Have sex with a male in the last 12 months** |  |  |
| No | 9894 (27.2%) | 5561 (24.6%) |
| Yes | 25495 (70.1%) | 16180 (71.5%) |
| Missing | 1004 (2.8%) | 874 (3.9%) |
| **Condoms use with male partners in the last 12 months** |  |  |
| Always | 5322 (14.6%) | 2949 (13.0%) |
| Never | 2680 (7.4%) | 2025 (9.0%) |
| Not applicable (no vag/anal sex) | 599 (1.6%) | 366 (1.6%) |
| Unknown or Declined to answer | 1298 (3.6%) | 762 (3.4%) |
| Usually (>50%) or Sometimes | 16291 (44.8%) | 10394 (46.0%) |
| Missing | 10203 (28.0%) | 6119 (27.1%) |
| **Have sex with a female in the last 12 months** |  |  |
| No | 23728 (65.2%) | 14972 (66.2%) |
| Yes | 11655 (32.0%) | 6751 (29.9%) |
| Missing | 1010 (2.8%) | 892 (3.9%) |
| **Condoms use with female partners in the last 12 months** |  |  |
| Always | 1694 (4.7%) | 1034 (4.6%) |
| Never | 1348 (3.7%) | 794 (3.5%) |
| Not applicable (no vag/anal sex) | 9604 (26.4%) | 7347 (32.5%) |
| Unknown or Declined to answer | 830 (2.3%) | 490 (2.2%) |
| Usually (>50%) or Sometimes | 6820 (18.7%) | 3909 (17.3%) |
| Missing | 16097 (44.2%) | 9041 (40.0%) |
| ***Drug injection*** |  |  |
| Less than 12 months ago | 120 (0.3%) | 102 (0.5%) |
| Less than 3 months ago | 352 (1.0%) | 268 (1.2%) |
| More than 12 months ago | 302 (0.8%) | 177 (0.8%) |
| Never injected | 34450 (94.7%) | 21231 (93.9%) |
| Missing | 1169 (3.2%) | 837 (3.7%) |
| **Pre-exposure prophylaxis (PrEP) use** |  |  |
| No | 34203 (94.0%) | 20350 (90.0%) |
| Yes | 2190 (6.0%) | 2265 (10.0%) |
| **Sex oversea (outside Australia or New Zealand) in the last 12 months** |  |  |
| No | 19108 (52.5%) | 14298 (63.2%) |
| Yes | 14064 (38.6%) | 6013 (26.6%) |
| Missing | 3221 (8.9%) | 2304 (10.2%) |
| **Current sex worker** |  |  |
| No | 33916 (93.2%) | 21872 (96.7%) |
| Yes | 2477 (6.8%) | 743 (3.3%) |
| **Contact with a gonorrhoea case** |  |  |
| No/Missing | 35671 (98.0%) | 21808 (96.4%) |
| Yes | 722 (2.0%) | 807 (3.6%) |
| **Contact with a chlamydia case** |  |  |
| No/Missing | 34980 (96.1%) | 21234 (93.9%) |
| Yes | 1413 (3.9%) | 1381 (6.1%) |
| **Contact with a syphilis case** |  |  |
| No/Missing | 36082 (99.1%) | 22260 (98.4%) |
| Yes | 311 (0.9%) | 355 (1.6%) |
| **STI symptom** |  |  |
| No | 21452 (58.9%) | 5437 (24.0%) |
| Yes | 11860 (32.6%) | 9406 (41.6%) |
| Missing | 3081 (8.5%) | 7772 (34.4%) |
| **Past genital warts infection** |  |  |
| No | 11252 (30.9%) | 7975 (35.3%) |
| Yes | 2113 (5.8%) | 1441 (6.4%) |
| Missing | 23028 (63.3%) | 13199 (58.4%) |
| **Past chlamydia infection** |  |  |
| No | 4922 (13.5%) | 3403 (15.0%) |
| Yes | 8443 (23.2%) | 6013 (26.6%) |
| Missing | 23028 (63.3%) | 13199 (58.4%) |
| **Past gonorrhoea infection** |  |  |
| No | 8242 (22.6%) | 5372 (23.8%) |
| Yes | 5123 (14.1%) | 4044 (17.9%) |
| Missing | 23028 (63.3%) | 13199 (58.4%) |
| **Past non-specific urethritis infection** |  |  |
| No | 9339 (25.7%) | 6814 (30.1%) |
| Yes | 282 (0.8%) | 226 (1.0%) |
| Missing | 26772 (73.6%) | 15575 (68.9%) |
| **Past syphilis infection** |  |  |
| No | 11494 (31.6%) | 7839 (34.7%) |
| Yes | 1871 (5.1%) | 1577 (7.0%) |
| Missing | 23028 (63.3%) | 13199 (58.4%) |
| **Past genital Herpes** |  |  |
| No | 12546 (34.5%) | 8774 (38.8%) |
| Yes | 1500 (4.1%) | 1058 (4.7%) |
| Missing | 22347 (61.4%) | 12783 (56.5%) |
| **HIV infection** |  |  |
| HIV-negative | 12851 (35.3%) | 8992 (39.8%) |
| Living with HIV | 514 (1.4%) | 424 (1.9%) |
| Missing | 23028 (63.3%) | 13199 (58.4%) |
| **Past Hepatitis B infection** |  |  |
| No | 13230 (36.4%) | 9330 (41.3%) |
| Yes | 135 (0.4%) | 86 (0.4%) |
| Missing | 23028 (63.3%) | 13199 (58.4%) |
| **Past Hepatitis C infection** |  |  |
| No | 13283 (36.5%) | 9366 (41.4%) |
| Yes | 82 (0.2%) | 50 (0.2%) |
| Missing | 23028 (63.3%) | 13199 (58.4%) |
| **Other past STI infections** |  |  |
| No | 13283 (36.5%) | 9366 (41.4%) |
| Yes | 82 (0.2%) | 50 (0.2%) |
| Missing | 23028 (63.3%) | 13199 (58.4%) |
| **Age at consultation** |  |  |
| Median (IQR) | 28.0(24.0-34.0) | 29.0(25.0-34.0) |
| **Number of casual male sexual partners in the last 12 months** |  |  |
| Median (IQR) | 4.0(2.0-8.0) | 4.0(2.0-8.0) |
| Missing (Not applicable (no male sexual partners) / Unknown or Declined to answer) | 11636 (32.0%) | 6870 (30.4%) |
| **Number of casual female sexual partners in the last 12 months** |  |  |
| Median (IQR) | 3.0(2.0-6.0) | 3.0(2.0-6.0) |
| Missing (Not applicable (no male sexual partners) / Unknown or Declined to answer) | 25124 (69.0%) | 16105 (71.2%) |

^a^ IQR: interquartile range

^b^ STI: sexually transmitted infections

**The stacking ensemble learning architectures**

Table S6. The stacking ensemble learning architectures

| **Stacking ensemble learning** | **Base models** |
| --- | --- |
| stacking with 2 base models | ENR+ GBM, ENR+ RF, ENR + NB, ENR + DL, GBM + RF, GBM + NB, GBM + DL, RF + NB, RF + DL, and NB + DL |
| stacking with 3 base models | ENR+ GBM+ RF, ENR+ GBM+ NB, ENR + GBM+ DL, ENR+ RF+ NB, and ENR+ RF+ DL; ENR+NB+DL; NB+DL+RF; NB+DL+GBM; NB+RF+GBM; DL+RF+GBM |
| stacking with 4 base models | ENR +GBM + RF + NB, ENR+ GBM+ RF+ DL, ENR+ GBM+ NB+ DL, ENR+ RF+ NB+ DL, and GBM +RF+ NB +DL |
| stacking with 5 base models | ENR+ GBM+ RF+ NB+ DL |

^a^ DL: deep learning (neural networks)

^b^ ENR: elastic net regression

^c^ GBM: gradient boosting machine

^d^ NB: naive Bayes

^e^ RF: random forest

Performance metrics of machine learning models on testing and external analysis

**Preliminary results of four machine learning regressions on testing and external analysis**

Based on the results of four regressions, we found elastic net regression was better than the other three regressions. Therefore, we selected elastic net regression as the base learner for ensemble learning.

Table S7. AUC of regression models for predicting HIV acquisition

| Models | Testing data  (2015-2018) | | | External validation data  (2019) | | | External validation data  (2020 - 2021) | | |
| --- | --- | --- | --- | --- | --- | --- | --- | --- | --- |
|  | Mean | 95%CI |  | Mean | 95%CI |  | Mean | 95%CI |  |
| LR | 0.7605 | 0.6952 | 0.8257 | 0.6765 | 0.6123 | 0.7407 | 0.5923 | 0.4735 | 0.7112 |
| LASSO | 0.7747 | 0.7131 | 0.8363 | 0.7909 | 0.7458 | 0.8361 | 0.6844 | 0.5817 | 0.7872 |
| RR | 0.7692 | 0.7049 | 0.8335 | 0.7257 | 0.6672 | 0.7842 | 0.6513 | 0.5425 | 0.7602 |
| ENR | 0.7764 | 0.7162 | 0.8367 | 0.7684 | 0.7195 | 0.8173 | 0.6816 | 0.5823 | 0.7809 |

^a^ CI: confidence interval

^b^ ENR: elastic net regression

^c^ LASSO: least absolute shrinkage and selection operator regression

^d^ LR: logistic regression

^e^ RR: ridge regression

Table S8. AUC of regression models for predicting syphilis acquisition

| Models | Testing data  (2015-2018) | | | External validation data  (2019) | | | External validation data  (2020 - 2021) | | |
| --- | --- | --- | --- | --- | --- | --- | --- | --- | --- |
|  | Mean | 95%CI |  | Mean | 95%CI |  | Mean | 95%CI |  |
| LR | 0.8377 | 0.8175 | 0.8579 | 0.8414 | 0.8271 | 0.8557 | 0.8228 | 0.8061 | 0.8395 |
| LASSO | 0.8380 | 0.8179 | 0.8582 | 0.8418 | 0.8275 | 0.8561 | 0.8232 | 0.8066 | 0.8398 |
| RR | 0.8378 | 0.8176 | 0.8579 | 0.8415 | 0.8272 | 0.8558 | 0.8229 | 0.8062 | 0.8395 |
| ENR | 0.8402 | 0.8205 | 0.8599 | 0.8443 | 0.8304 | 0.8583 | 0.8269 | 0.8111 | 0.8428 |

^a^ CI: confidence interval

^b^ ENR: elastic net regression

^c^ LASSO: least absolute shrinkage and selection operator regression

^d^ LR: logistic regression

^e^ RR: ridge regression

Table S9. AUC of regression models for predicting gonorrhoea acquisition

| Models | Testing data  (2015-2018) | | | External validation data  (2019) | | | External validation data  (2020 - 2021) | | |
| --- | --- | --- | --- | --- | --- | --- | --- | --- | --- |
|  | Mean | 95%CI |  | Mean | 95%CI |  | Mean | 95%CI |  |
| LR | 0.7655 | 0.7541 | 0.7770 | 0.8031 | 0.7949 | 0.8112 | 0.7870 | 0.7767 | 0.7974 |
| LASSO | 0.7656 | 0.7542 | 0.7771 | 0.8028 | 0.7947 | 0.8109 | 0.7867 | 0.7764 | 0.7971 |
| RR | 0.7655 | 0.7541 | 0.7770 | 0.8030 | 0.7949 | 0.8112 | 0.7870 | 0.7766 | 0.7974 |
| ENR | 0.7656 | 0.7542 | 0.7771 | 0.8024 | 0.7942 | 0.8105 | 0.7863 | 0.7760 | 0.7967 |

^a^ CI: confidence interval

^b^ ENR: elastic net regression

^c^ LASSO: least absolute shrinkage and selection operator regression

^d^ LR: logistic regression

^e^ RR: ridge regression

Table S10. AUC of regression models for predicting chlamydia acquisition

| Models | Testing data  (2015-2018) | | | External validation data  (2019) | | | External validation data  (2020 - 2021) | | |
| --- | --- | --- | --- | --- | --- | --- | --- | --- | --- |
|  | Mean | 95%CI | | Mean | 95%CI | | Mean | 95%CI | |
| LR | 0.6920 | 0.6801 | 0.7039 | 0.6852 | 0.6756 | 0.6948 | 0.6925 | 0.6808 | 0.7043 |
| LASSO | 0.6921 | 0.6802 | 0.7040 | 0.6854 | 0.6757 | 0.6950 | 0.6926 | 0.6808 | 0.7043 |
| RR | 0.6920 | 0.6801 | 0.7039 | 0.6852 | 0.6756 | 0.6948 | 0.6925 | 0.6808 | 0.7043 |
| ENR | 0.6920 | 0.6801 | 0.7039 | 0.6855 | 0.6759 | 0.6951 | 0.6924 | 0.6807 | 0.7042 |

^a^ CI: confidence interval

^b^ ENR: elastic net regression

^c^ LASSO: least absolute shrinkage and selection operator regression

^d^ LR: logistic regression

^e^ RR: ridge regression

**Acceptable or excellent performance on testing analysis and stable performance on external validation analysis**

Our best models provided acceptable or excellent performance on testing for predicting HIV (ENR+GBM+RF, AUC= 0.783), syphilis (ENR+GBM+RF, AUC = 0.844), gonorrhoea (ENR+GBM+RF, AUC= 0.779), and chlamydia (ENR+GBM+RF, AUC = 0.704).

Figure S1. Receiver operating characteristic curves for testing to detect HIV, syphilis, gonorrhoea, and chlamydia. DL: deep learning (neural networks); ENR: elastic net regression; GBM: gradient boosting machine; NB: naive Bayes; RF: random forest.


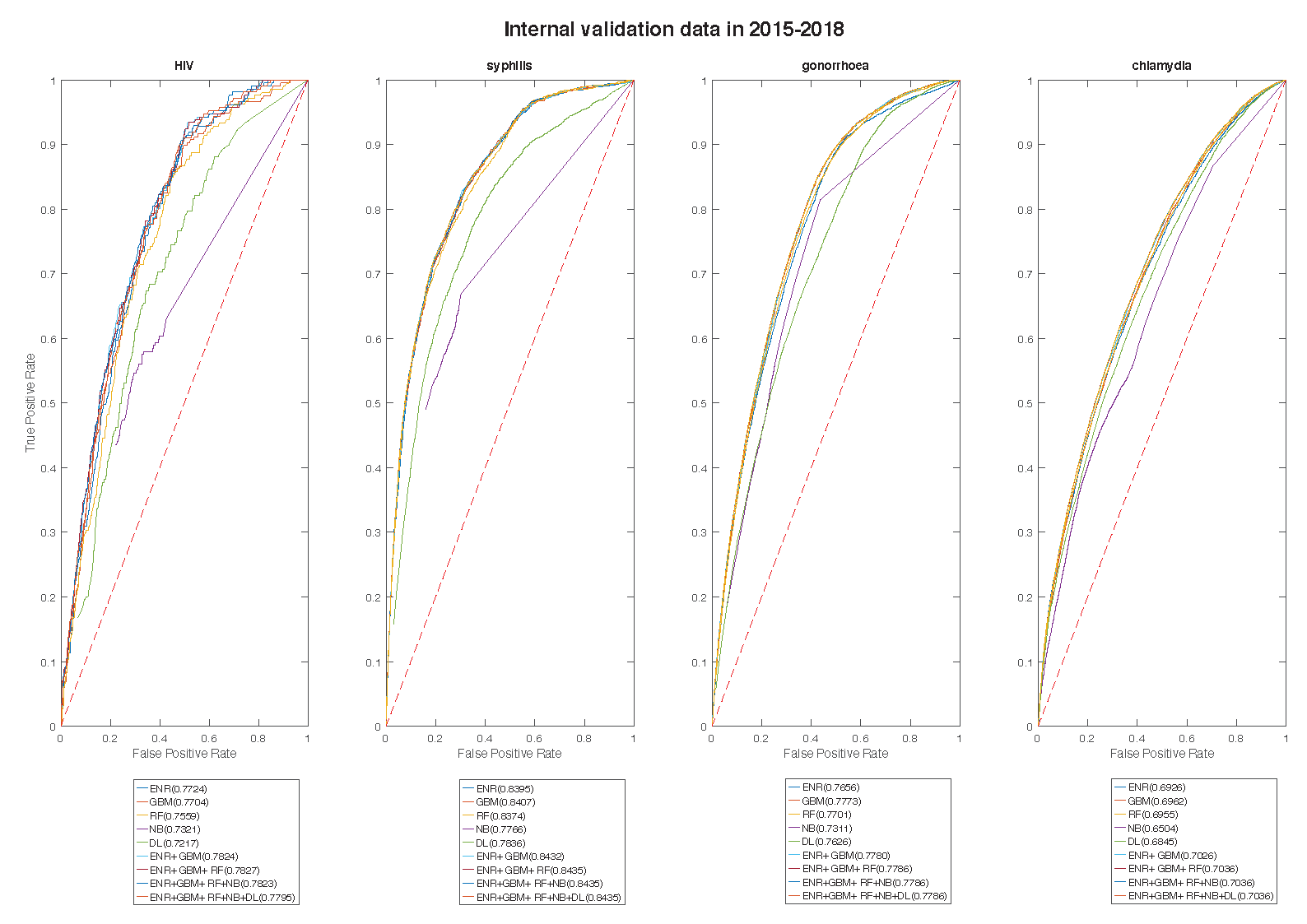


Our external validation results showed very comparable AUC to the testing. Our models provided acceptable or excellent performance in external validation for predicting HIV (ENR+GBM+RF, AUC=0.796 in 2019; AUC=0.702 in 2020 and 2021), syphilis (ENR+GBM+RF, AUC=0.848 in 2019; AUC=0.834 in 2020 and 2021), gonorrhoea (ENR+GBM+RF, AUC=0.811 in 2019; AUC=0.791 in 2020 and 2021), and chlamydia (ENR+GBM+RF, AUC=0.691 in 2019 and AUC=0.688 in 2020 and 2021). (Figure S3). We also provided the external validation results of 34 models in the supplemental materials. (Tables S10-21).

Figure S2. Receiver operating characteristic curves for external validation to detect HIV, syphilis, gonorrhoea, and chlamydia. DL: deep learning (neural networks); ENR: elastic net regression; GBM: gradient boosting machine; NB: naive Bayes; RF: random forest.


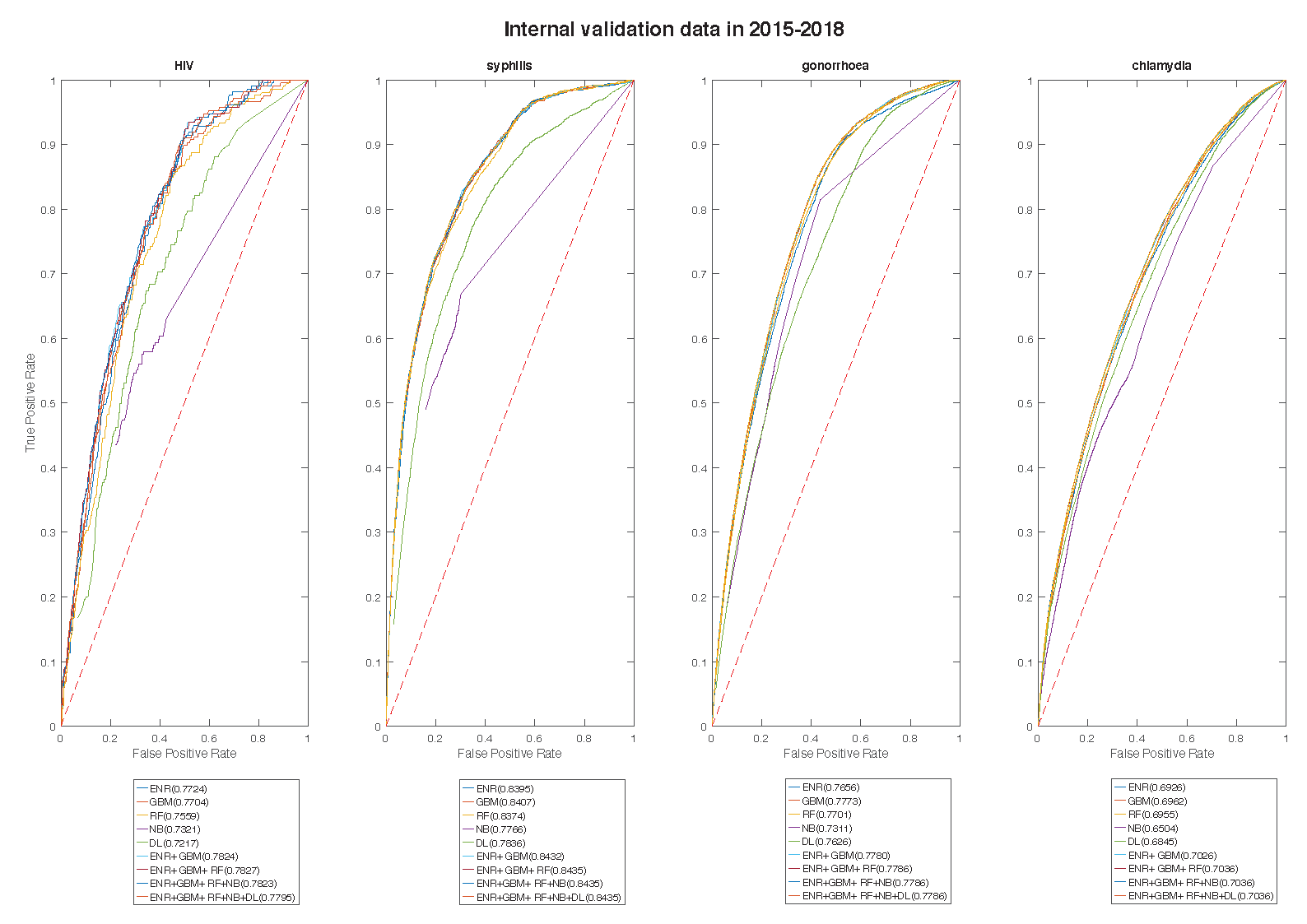


Supplementary Table 11. AUC of machine learning models for predicting HIV acquisition

| Models | Testing data  (2015-2018) | | | External validation data  (2019) | | | External validation data  (2020 - 2021) | | |
| --- | --- | --- | --- | --- | --- | --- | --- | --- | --- |
|  | Mean | 95%CI | | Mean | 95%CI | | Mean | 95%CI | |
| ENR | 0.7724 | 0.7279 | 0.8170 | 0.7627 | 0.7083 | 0.8171 | 0.6700 | 0.5717 | 0.7683 |
| GBM | 0.7704 | 0.7223 | 0.8185 | 0.7823 | 0.7389 | 0.8257 | 0.7105 | 0.6222 | 0.7989 |
| RF | 0.7559 | 0.7092 | 0.8027 | 0.7707 | 0.7225 | 0.8188 | 0.6738 | 0.5822 | 0.7655 |
| NB | 0.7321 | 0.6847 | 0.7794 | 0.7721 | 0.7257 | 0.8184 | 0.6336 | 0.5318 | 0.7353 |
| DL | 0.7217 | 0.6713 | 0.7722 | 0.7044 | 0.6455 | 0.7632 | 0.6500 | 0.5525 | 0.7475 |
| ENR+ GBM | 0.7824 | 0.7369 | 0.8278 | 0.7925 | 0.7478 | 0.8373 | 0.7006 | 0.6031 | 0.7982 |
| ENR +RF | 0.7746 | 0.7298 | 0.8194 | 0.7760 | 0.7244 | 0.8275 | 0.6786 | 0.5842 | 0.7731 |
| ENR + NB | 0.7734 | 0.7285 | 0.8183 | 0.7745 | 0.7236 | 0.8253 | 0.6783 | 0.5807 | 0.7758 |
| ENR + DL | 0.7694 | 0.7253 | 0.8135 | 0.7572 | 0.7016 | 0.8127 | 0.6710 | 0.5718 | 0.7702 |
| GBM + RF | 0.7768 | 0.7304 | 0.8233 | 0.7939 | 0.7510 | 0.8369 | 0.7062 | 0.6184 | 0.7941 |
| GBM + NB | 0.7695 | 0.7217 | 0.8173 | 0.7789 | 0.7352 | 0.8225 | 0.7001 | 0.6074 | 0.7928 |
| GBM + DL | 0.7685 | 0.7225 | 0.8145 | 0.7827 | 0.7391 | 0.8262 | 0.7051 | 0.6144 | 0.7959 |
| RF + NB | 0.7596 | 0.7125 | 0.8067 | 0.7796 | 0.7328 | 0.8265 | 0.6723 | 0.5771 | 0.7675 |
| RF + DL | 0.7598 | 0.7147 | 0.8048 | 0.7696 | 0.7218 | 0.8173 | 0.6784 | 0.5875 | 0.7693 |
| NB + DL | 0.7301 | 0.6809 | 0.7793 | 0.7521 | 0.7025 | 0.8017 | 0.6688 | 0.5721 | 0.7655 |
| ENR+ GBM+ RF | 0.7827 | 0.7375 | 0.8280 | 0.7958 | 0.7512 | 0.8403 | 0.7015 | 0.6062 | 0.7969 |
| ENR+ GBM+ NB | 0.7821 | 0.7364 | 0.8277 | 0.7918 | 0.7471 | 0.8366 | 0.6976 | 0.5988 | 0.7965 |
| ENR+GBM+ DL | 0.7796 | 0.7350 | 0.8242 | 0.7892 | 0.7438 | 0.8345 | 0.7003 | 0.6025 | 0.7981 |
| ENR+ RF+ NB | 0.7750 | 0.7298 | 0.8202 | 0.7828 | 0.7336 | 0.8320 | 0.6809 | 0.5861 | 0.7758 |
| ENR+ RF+ DL | 0.7723 | 0.7282 | 0.8165 | 0.7708 | 0.7186 | 0.8229 | 0.6784 | 0.5832 | 0.7736 |
| ENR+ NB + DL | 0.7701 | 0.7257 | 0.8144 | 0.7686 | 0.7165 | 0.8207 | 0.6793 | 0.5808 | 0.7779 |
| GBM+ RF + NB | 0.7750 | 0.7280 | 0.8220 | 0.7906 | 0.7470 | 0.8341 | 0.6976 | 0.6066 | 0.7886 |
| GBM + RF + DL | 0.7752 | 0.7308 | 0.8196 | 0.7916 | 0.7480 | 0.8353 | 0.7036 | 0.6156 | 0.7916 |
| GBM + NB + DL | 0.7678 | 0.7219 | 0.8137 | 0.7803 | 0.7358 | 0.8247 | 0.7003 | 0.6060 | 0.7945 |
| RF + NB +DL | 0.7631 | 0.7181 | 0.8081 | 0.7772 | 0.7300 | 0.8243 | 0.6759 | 0.5819 | 0.7699 |
| ENR+GBM+ RF+NB | 0.7823 | 0.7368 | 0.8277 | 0.7951 | 0.7506 | 0.8396 | 0.6986 | 0.6022 | 0.7951 |
| ENR+GBM+ RF+DL | 0.7802 | 0.7359 | 0.8246 | 0.7923 | 0.7472 | 0.8373 | 0.7009 | 0.6052 | 0.7966 |
| ENR+GBM+NB+DL | 0.7790 | 0.7341 | 0.8238 | 0.7880 | 0.7426 | 0.8335 | 0.6969 | 0.5976 | 0.7961 |
| ENR+RF+NB+DL | 0.7725 | 0.7280 | 0.8170 | 0.7770 | 0.7270 | 0.8270 | 0.6800 | 0.5843 | 0.7757 |
| GBM+ RF+NB+DL | 0.7725 | 0.7280 | 0.8170 | 0.7770 | 0.7270 | 0.8270 | 0.6800 | 0.5843 | 0.7757 |
| ENR+GBM+ RF+NB+DL | 0.7795 | 0.7349 | 0.8240 | 0.7911 | 0.7460 | 0.8363 | 0.6975 | 0.6005 | 0.7944 |

^a^ AUC: areas under the curve

^b^ CI: confidence interval

^c^ DL: deep learning (neural networks)

^d^ ENR: elastic net regression

^e^ GBM: gradient boosting machine

^f^ NB: naive Bayes

^g^ RF: random forest

Supplementary Table 12. Sensitivity of machine learning models for predicting HIV acquisition

| Models | Testing data  (2015-2018) | | | External validation data  (2019) | | | External validation data  (2020 - 2021) | | |
| --- | --- | --- | --- | --- | --- | --- | --- | --- | --- |
|  | Mean | 95%CI | | Mean | 95%CI | | Mean | 95%CI | |
| ENR | 0.6914 | 0.6861 | 0.6967 | 0.8426 | 0.8385 | 0.8467 | 0.8402 | 0.8350 | 0.8454 |
| GBM | 0.7664 | 0.7616 | 0.7711 | 0.8013 | 0.7968 | 0.8059 | 0.7775 | 0.7715 | 0.7834 |
| RF | 0.7513 | 0.7464 | 0.7562 | 0.8016 | 0.7971 | 0.8060 | 0.7879 | 0.7821 | 0.7936 |
| NB | 0.6243 | 0.6188 | 0.6298 | 0.7905 | 0.7859 | 0.7950 | 0.7704 | 0.7645 | 0.7763 |
| DL | 0.7664 | 0.7616 | 0.7712 | 0.8355 | 0.8314 | 0.8395 | 0.8203 | 0.8151 | 0.8255 |
| ENR+ GBM | 0.7501 | 0.7452 | 0.7551 | 0.8750 | 0.8712 | 0.8788 | 0.8710 | 0.8661 | 0.8758 |
| ENR +RF | 0.7162 | 0.7111 | 0.7213 | 0.8545 | 0.8505 | 0.8585 | 0.8521 | 0.8470 | 0.8571 |
| ENR + NB | 0.6941 | 0.6889 | 0.6994 | 0.8458 | 0.8417 | 0.8498 | 0.8442 | 0.8391 | 0.8493 |
| ENR + DL | 0.7302 | 0.7252 | 0.7353 | 0.8588 | 0.8548 | 0.8627 | 0.8535 | 0.8485 | 0.8585 |
| GBM + RF | 0.7771 | 0.7723 | 0.7818 | 0.8303 | 0.8260 | 0.8347 | 0.8121 | 0.8064 | 0.8178 |
| GBM + NB | 0.7607 | 0.7559 | 0.7656 | 0.8351 | 0.8308 | 0.8394 | 0.8222 | 0.8166 | 0.8278 |
| GBM + DL | 0.8316 | 0.8274 | 0.8358 | 0.8949 | 0.8914 | 0.8984 | 0.8905 | 0.8860 | 0.8950 |
| RF + NB | 0.7269 | 0.7218 | 0.7320 | 0.7963 | 0.7918 | 0.8009 | 0.7840 | 0.7781 | 0.7898 |
| RF + DL | 0.7849 | 0.7803 | 0.7896 | 0.8410 | 0.8370 | 0.8450 | 0.8264 | 0.8212 | 0.8316 |
| NB + DL | 0.7573 | 0.7525 | 0.7622 | 0.8404 | 0.8364 | 0.8443 | 0.8265 | 0.8214 | 0.8315 |
| ENR+ GBM+ RF | 0.7514 | 0.7465 | 0.7563 | 0.8731 | 0.8693 | 0.8770 | 0.8681 | 0.8633 | 0.8730 |
| ENR+ GBM+ NB | 0.7521 | 0.7472 | 0.7570 | 0.8777 | 0.8739 | 0.8814 | 0.8747 | 0.8699 | 0.8795 |
| ENR+GBM+ DL | 0.7816 | 0.7769 | 0.7862 | 0.8869 | 0.8833 | 0.8905 | 0.8823 | 0.8777 | 0.8869 |
| ENR+ RF+ NB | 0.7179 | 0.7128 | 0.7230 | 0.8570 | 0.8531 | 0.8610 | 0.8551 | 0.8501 | 0.8601 |
| ENR+ RF+ DL | 0.7450 | 0.7401 | 0.7499 | 0.8652 | 0.8613 | 0.8690 | 0.8615 | 0.8567 | 0.8664 |
| ENR+ NB + DL | 0.7337 | 0.7287 | 0.7387 | 0.8616 | 0.8577 | 0.8654 | 0.8571 | 0.8522 | 0.8620 |
| GBM+ RF + NB | 0.7574 | 0.7526 | 0.7623 | 0.8289 | 0.8245 | 0.8332 | 0.8126 | 0.8070 | 0.8183 |
| GBM + RF + DL | 0.8134 | 0.8089 | 0.8178 | 0.8737 | 0.8699 | 0.8774 | 0.8644 | 0.8595 | 0.8692 |
| GBM + NB + DL | 0.8174 | 0.8130 | 0.8217 | 0.8888 | 0.8853 | 0.8924 | 0.8840 | 0.8795 | 0.8886 |
| RF + NB +DL | 0.7702 | 0.7654 | 0.7750 | 0.8377 | 0.8336 | 0.8417 | 0.8247 | 0.8194 | 0.8299 |
| ENR+GBM+ RF+NB | 0.7526 | 0.7477 | 0.7575 | 0.8749 | 0.8712 | 0.8787 | 0.8708 | 0.8660 | 0.8756 |
| ENR+GBM+ RF+DL | 0.7801 | 0.7754 | 0.7848 | 0.8823 | 0.8786 | 0.8860 | 0.8774 | 0.8728 | 0.8821 |
| ENR+GBM+NB+DL | 0.7842 | 0.7795 | 0.7889 | 0.8892 | 0.8857 | 0.8928 | 0.8852 | 0.8807 | 0.8897 |
| ENR+RF+NB+DL | 0.7457 | 0.7408 | 0.7506 | 0.8658 | 0.8620 | 0.8697 | 0.8626 | 0.8578 | 0.8675 |
| GBM+ RF+NB+DL | 0.7457 | 0.7408 | 0.7506 | 0.8658 | 0.8620 | 0.8697 | 0.8626 | 0.8578 | 0.8675 |
| ENR+GBM+ RF+NB+DL | 0.7827 | 0.7780 | 0.7874 | 0.8842 | 0.8806 | 0.8879 | 0.8801 | 0.8755 | 0.8847 |

^a^ CI: confidence interval

^b^ DL: deep learning (neural networks)

^c^ ENR: elastic net regression

^d^ GBM: gradient boosting machine

^e^ NB: naive Bayes

^f^ RF: random forest

Supplementary Table 13. Specificity of machine learning models for predicting HIV acquisition

| Models | Testing data  (2015-2018) | | | External validation data  (2019) | | | External validation data  (2020 - 2021) | | |
| --- | --- | --- | --- | --- | --- | --- | --- | --- | --- |
|  | Mean | 95%CI | | Mean | 95%CI | | Mean | 95%CI | |
| ENR | 0.6968 | 0.5916 | 0.8020 | 0.4211 | 0.3020 | 0.5401 | 0.8402 | 0.8350 | 0.8454 |
| GBM | 0.6161 | 0.5070 | 0.7252 | 0.5556 | 0.4355 | 0.6756 | 0.7775 | 0.7715 | 0.7834 |
| RF | 0.6125 | 0.5015 | 0.7235 | 0.5322 | 0.4155 | 0.6489 | 0.7879 | 0.7821 | 0.7936 |
| NB | 0.7862 | 0.6918 | 0.8806 | 0.4678 | 0.3396 | 0.5960 | 0.7704 | 0.7645 | 0.7763 |
| DL | 0.4992 | 0.3918 | 0.6066 | 0.3860 | 0.2692 | 0.5027 | 0.8203 | 0.8151 | 0.8255 |
| ENR+ GBM | 0.6577 | 0.5480 | 0.7673 | 0.3567 | 0.2358 | 0.4777 | 0.8710 | 0.8661 | 0.8758 |
| ENR +RF | 0.6800 | 0.5727 | 0.7873 | 0.4094 | 0.2881 | 0.5306 | 0.8521 | 0.8470 | 0.8571 |
| ENR + NB | 0.7017 | 0.5967 | 0.8066 | 0.4211 | 0.3020 | 0.5401 | 0.8442 | 0.8391 | 0.8493 |
| ENR + DL | 0.6495 | 0.5423 | 0.7567 | 0.4035 | 0.2882 | 0.5188 | 0.8535 | 0.8485 | 0.8585 |
| GBM + RF | 0.6188 | 0.5076 | 0.7300 | 0.5029 | 0.3744 | 0.6314 | 0.8121 | 0.8064 | 0.8178 |
| GBM + NB | 0.6209 | 0.5121 | 0.7297 | 0.4561 | 0.3292 | 0.5831 | 0.8222 | 0.8166 | 0.8278 |
| GBM + DL | 0.4591 | 0.3471 | 0.5712 | 0.2924 | 0.1752 | 0.4096 | 0.2963 | 0.1343 | 0.4583 |
| RF + NB | 0.6511 | 0.5411 | 0.7612 | 0.5614 | 0.4408 | 0.6820 | 0.4198 | 0.2633 | 0.5762 |
| RF + DL | 0.5436 | 0.4334 | 0.6539 | 0.4737 | 0.3607 | 0.5867 | 0.3210 | 0.1985 | 0.4435 |
| NB + DL | 0.5186 | 0.4095 | 0.6277 | 0.3860 | 0.2692 | 0.5027 | 0.2840 | 0.1710 | 0.3969 |
| ENR+ GBM+ RF | 0.6360 | 0.5252 | 0.7468 | 0.3626 | 0.2414 | 0.4837 | 0.2716 | 0.1188 | 0.4244 |
| ENR+ GBM+ NB | 0.6490 | 0.5388 | 0.7592 | 0.3509 | 0.2301 | 0.4716 | 0.2716 | 0.1188 | 0.4244 |
| ENR+GBM+ DL | 0.5915 | 0.4792 | 0.7038 | 0.3392 | 0.2220 | 0.4564 | 0.2593 | 0.1156 | 0.4029 |
| ENR+ RF+ NB | 0.6757 | 0.5680 | 0.7834 | 0.4035 | 0.2833 | 0.5237 | 0.2469 | 0.1081 | 0.3858 |
| ENR+ RF+ DL | 0.6322 | 0.5233 | 0.7411 | 0.3860 | 0.2679 | 0.5041 | 0.2346 | 0.0961 | 0.3731 |
| ENR+ NB + DL | 0.6447 | 0.5375 | 0.7519 | 0.4035 | 0.2882 | 0.5188 | 0.2593 | 0.1204 | 0.3981 |
| GBM+ RF + NB | 0.6430 | 0.5345 | 0.7514 | 0.4971 | 0.3701 | 0.6240 | 0.4198 | 0.2370 | 0.6025 |
| GBM + RF + DL | 0.5344 | 0.4205 | 0.6483 | 0.4211 | 0.2983 | 0.5438 | 0.2840 | 0.1370 | 0.4309 |
| GBM + NB + DL | 0.4591 | 0.3471 | 0.5712 | 0.3158 | 0.1989 | 0.4326 | 0.2840 | 0.1366 | 0.4313 |
| RF + NB +DL | 0.5678 | 0.4562 | 0.6794 | 0.4854 | 0.3695 | 0.6012 | 0.3210 | 0.1985 | 0.4435 |
| ENR+GBM+ RF+NB | 0.6360 | 0.5252 | 0.7468 | 0.3567 | 0.2358 | 0.4777 | 0.2716 | 0.1188 | 0.4244 |
| ENR+GBM+ RF+DL | 0.5785 | 0.4657 | 0.6914 | 0.3509 | 0.2333 | 0.4685 | 0.2716 | 0.1276 | 0.4156 |
| ENR+GBM+NB+DL | 0.5829 | 0.4702 | 0.6956 | 0.3333 | 0.2163 | 0.4503 | 0.2593 | 0.1156 | 0.4029 |
| ENR+RF+NB+DL | 0.6231 | 0.5138 | 0.7323 | 0.3860 | 0.2695 | 0.5024 | 0.2346 | 0.0961 | 0.3731 |
| GBM+ RF+NB+DL | 0.6231 | 0.5138 | 0.7323 | 0.3860 | 0.2695 | 0.5024 | 0.2346 | 0.0961 | 0.3731 |
| ENR+GBM+ RF+NB+DL | 0.5742 | 0.4612 | 0.6872 | 0.3450 | 0.2276 | 0.4624 | 0.2716 | 0.1276 | 0.4156 |

^a^ CI: confidence interval

^b^ DL: deep learning (neural networks)

^c^ ENR: elastic net regression

^d^ GBM: gradient boosting machine

^e^ NB: naive Bayes

^f^ RF: random forest

Supplementary Table 14. AUC of machine learning models for predicting syphilis acquisition

| Models | Testing data  (2015-2018) | | | External validation data  (2019) | | | External validation data  (2020 - 2021) | | |
| --- | --- | --- | --- | --- | --- | --- | --- | --- | --- |
|  | Mean | 95%CI | | Mean | 95%CI | | Mean | 95%CI | |
| ENR | 0.8395 | 0.8241 | 0.8548 | 0.8433 | 0.8293 | 0.8573 | 0.8276 | 0.8119 | 0.8434 |
| GBM | 0.8407 | 0.8254 | 0.8559 | 0.8435 | 0.8293 | 0.8577 | 0.8326 | 0.8171 | 0.8482 |
| RF | 0.8374 | 0.8219 | 0.8528 | 0.8422 | 0.8280 | 0.8564 | 0.8221 | 0.8059 | 0.8383 |
| NB | 0.7766 | 0.7593 | 0.7939 | 0.7885 | 0.7722 | 0.8047 | 0.7600 | 0.7419 | 0.7782 |
| DL | 0.7836 | 0.7648 | 0.8023 | 0.7706 | 0.7515 | 0.7898 | 0.7436 | 0.7219 | 0.7653 |
| ENR+ GBM | 0.8432 | 0.8281 | 0.8583 | 0.8465 | 0.8327 | 0.8603 | 0.8337 | 0.8183 | 0.8490 |
| ENR +RF | 0.8421 | 0.8270 | 0.8572 | 0.8468 | 0.8331 | 0.8606 | 0.8301 | 0.8146 | 0.8457 |
| ENR + NB | 0.8395 | 0.8241 | 0.8548 | 0.8433 | 0.8293 | 0.8573 | 0.8276 | 0.8119 | 0.8434 |
| ENR + DL | 0.8396 | 0.8243 | 0.8550 | 0.8433 | 0.8293 | 0.8574 | 0.8269 | 0.8111 | 0.8428 |
| GBM + RF | 0.8419 | 0.8267 | 0.8571 | 0.8456 | 0.8317 | 0.8596 | 0.8325 | 0.8169 | 0.8480 |
| GBM + NB | 0.8406 | 0.8254 | 0.8558 | 0.8437 | 0.8295 | 0.8579 | 0.8327 | 0.8173 | 0.8482 |
| GBM + DL | 0.8406 | 0.8253 | 0.8559 | 0.8444 | 0.8303 | 0.8585 | 0.8327 | 0.8171 | 0.8482 |
| RF + NB | 0.8373 | 0.8218 | 0.8527 | 0.8423 | 0.8281 | 0.8564 | 0.8223 | 0.8061 | 0.8384 |
| RF + DL | 0.8378 | 0.8224 | 0.8533 | 0.8427 | 0.8285 | 0.8568 | 0.8229 | 0.8067 | 0.8391 |
| NB + DL | 0.7856 | 0.7677 | 0.8034 | 0.7855 | 0.7678 | 0.8031 | 0.7603 | 0.7402 | 0.7804 |
| ENR+ GBM+ RF | 0.8435 | 0.8285 | 0.8586 | 0.8476 | 0.8338 | 0.8613 | 0.8336 | 0.8183 | 0.8490 |
| ENR+ GBM+ NB | 0.8432 | 0.8281 | 0.8583 | 0.8465 | 0.8327 | 0.8603 | 0.8337 | 0.8183 | 0.8490 |
| ENR+GBM+ DL | 0.8430 | 0.8279 | 0.8582 | 0.8467 | 0.8329 | 0.8605 | 0.8332 | 0.8177 | 0.8486 |
| ENR+ RF+ NB | 0.8421 | 0.8270 | 0.8572 | 0.8468 | 0.8331 | 0.8606 | 0.8301 | 0.8146 | 0.8456 |
| ENR+ RF+ DL | 0.8421 | 0.8270 | 0.8573 | 0.8468 | 0.8330 | 0.8605 | 0.8294 | 0.8138 | 0.8451 |
| ENR+ NB + DL | 0.8396 | 0.8243 | 0.8550 | 0.8433 | 0.8293 | 0.8574 | 0.8269 | 0.8111 | 0.8428 |
| GBM+ RF + NB | 0.8419 | 0.8267 | 0.8571 | 0.8457 | 0.8318 | 0.8597 | 0.8326 | 0.8171 | 0.8481 |
| GBM + RF + DL | 0.8418 | 0.8265 | 0.8570 | 0.8461 | 0.8322 | 0.8601 | 0.8323 | 0.8167 | 0.8479 |
| GBM + NB + DL | 0.8405 | 0.8251 | 0.8558 | 0.8446 | 0.8305 | 0.8587 | 0.8328 | 0.8173 | 0.8482 |
| RF + NB +DL | 0.8378 | 0.8223 | 0.8532 | 0.8428 | 0.8286 | 0.8569 | 0.8230 | 0.8069 | 0.8392 |
| ENR+GBM+ RF+NB | 0.8435 | 0.8285 | 0.8586 | 0.8476 | 0.8338 | 0.8613 | 0.8336 | 0.8183 | 0.8490 |
| ENR+GBM+ RF+DL | 0.8435 | 0.8284 | 0.8586 | 0.8476 | 0.8338 | 0.8613 | 0.8330 | 0.8176 | 0.8485 |
| ENR+GBM+NB+DL | 0.8430 | 0.8279 | 0.8582 | 0.8467 | 0.8329 | 0.8605 | 0.8331 | 0.8177 | 0.8486 |
| ENR+RF+NB+DL | 0.8421 | 0.8270 | 0.8573 | 0.8468 | 0.8330 | 0.8605 | 0.8294 | 0.8138 | 0.8451 |
| GBM+ RF+NB+DL | 0.8421 | 0.8270 | 0.8573 | 0.8468 | 0.8330 | 0.8605 | 0.8294 | 0.8138 | 0.8451 |
| ENR+GBM+ RF+NB+DL | 0.8435 | 0.8284 | 0.8586 | 0.8476 | 0.8338 | 0.8613 | 0.8330 | 0.8176 | 0.8485 |

^a^ AUC: areas under the curve

^b^ CI: confidence interval

^c^ DL: deep learning (neural networks)

^d^ ENR: elastic net regression

^e^ GBM: gradient boosting machine

^f^ NB: naive Bayes

^g^ RF: random forest

Supplementary Table 15. Sensitivity of machine learning models for predicting syphilis acquisition

| Models | Testing data  (2015-2018) | | | External validation data  (2019) | | | External validation data  (2020 - 2021) | | |
| --- | --- | --- | --- | --- | --- | --- | --- | --- | --- |
|  | Mean | 95%CI | | Mean | 95%CI | | Mean | 95%CI | |
| ENR | 0.7241 | 0.7191 | 0.7292 | 0.7760 | 0.7713 | 0.7807 | 0.7160 | 0.7096 | 0.7225 |
| GBM | 0.7513 | 0.7465 | 0.7562 | 0.7886 | 0.7839 | 0.7932 | 0.7433 | 0.7370 | 0.7496 |
| RF | 0.7790 | 0.7743 | 0.7836 | 0.8007 | 0.7962 | 0.8052 | 0.7360 | 0.7297 | 0.7423 |
| NB | 0.5971 | 0.5916 | 0.6026 | 0.6302 | 0.6248 | 0.6357 | 0.5549 | 0.5478 | 0.5620 |
| DL | 0.7799 | 0.7752 | 0.7846 | 0.8295 | 0.8252 | 0.8337 | 0.7748 | 0.7688 | 0.7808 |
| ENR+ GBM | 0.7425 | 0.7376 | 0.7474 | 0.7872 | 0.7825 | 0.7918 | 0.7337 | 0.7274 | 0.7400 |
| ENR +RF | 0.7516 | 0.7468 | 0.7565 | 0.7928 | 0.7882 | 0.7974 | 0.7373 | 0.7310 | 0.7436 |
| ENR + NB | 0.7241 | 0.7191 | 0.7292 | 0.7760 | 0.7713 | 0.7807 | 0.7160 | 0.7096 | 0.7225 |
| ENR + DL | 0.7422 | 0.7373 | 0.7472 | 0.7937 | 0.7891 | 0.7983 | 0.7374 | 0.7312 | 0.7437 |
| GBM + RF | 0.7697 | 0.7650 | 0.7745 | 0.8024 | 0.7979 | 0.8070 | 0.7493 | 0.7431 | 0.7555 |
| GBM + NB | 0.7531 | 0.7482 | 0.7579 | 0.7924 | 0.7878 | 0.7971 | 0.7435 | 0.7372 | 0.7497 |
| GBM + DL | 0.7625 | 0.7577 | 0.7673 | 0.8039 | 0.7994 | 0.8084 | 0.7566 | 0.7504 | 0.7627 |
| RF + NB | 0.7794 | 0.7747 | 0.7840 | 0.8015 | 0.7970 | 0.8060 | 0.7366 | 0.7303 | 0.7429 |
| RF + DL | 0.7951 | 0.7905 | 0.7996 | 0.8201 | 0.8157 | 0.8245 | 0.7665 | 0.7604 | 0.7726 |
| NB + DL | 0.7283 | 0.7233 | 0.7333 | 0.7582 | 0.7534 | 0.7631 | 0.6950 | 0.6885 | 0.7016 |
| ENR+ GBM+ RF | 0.7578 | 0.7529 | 0.7626 | 0.7971 | 0.7925 | 0.8017 | 0.7436 | 0.7374 | 0.7499 |
| ENR+ GBM+ NB | 0.7423 | 0.7374 | 0.7472 | 0.7870 | 0.7824 | 0.7917 | 0.7335 | 0.7272 | 0.7399 |
| ENR+GBM+ DL | 0.7557 | 0.7509 | 0.7606 | 0.8006 | 0.7961 | 0.8052 | 0.7477 | 0.7415 | 0.7539 |
| ENR+ RF+ NB | 0.7516 | 0.7468 | 0.7565 | 0.7929 | 0.7882 | 0.7975 | 0.7374 | 0.7310 | 0.7437 |
| ENR+ RF+ DL | 0.7671 | 0.7624 | 0.7719 | 0.8065 | 0.8020 | 0.8110 | 0.7553 | 0.7492 | 0.7615 |
| ENR+ NB + DL | 0.7422 | 0.7373 | 0.7471 | 0.7937 | 0.7891 | 0.7983 | 0.7375 | 0.7312 | 0.7438 |
| GBM+ RF + NB | 0.7697 | 0.7650 | 0.7744 | 0.8042 | 0.7996 | 0.8087 | 0.7497 | 0.7435 | 0.7559 |
| GBM + RF + DL | 0.7811 | 0.7765 | 0.7858 | 0.8159 | 0.8115 | 0.8203 | 0.7650 | 0.7589 | 0.7711 |
| GBM + NB + DL | 0.7635 | 0.7587 | 0.7683 | 0.8059 | 0.8014 | 0.8103 | 0.7570 | 0.7509 | 0.7632 |
| RF + NB +DL | 0.7954 | 0.7909 | 0.8000 | 0.8207 | 0.8164 | 0.8251 | 0.7671 | 0.7610 | 0.7731 |
| ENR+GBM+ RF+NB | 0.7579 | 0.7530 | 0.7627 | 0.7972 | 0.7927 | 0.8018 | 0.7437 | 0.7374 | 0.7499 |
| ENR+GBM+ RF+DL | 0.7678 | 0.7630 | 0.7725 | 0.8070 | 0.8025 | 0.8115 | 0.7550 | 0.7489 | 0.7612 |
| ENR+GBM+NB+DL | 0.7557 | 0.7509 | 0.7606 | 0.8006 | 0.7961 | 0.8052 | 0.7476 | 0.7414 | 0.7539 |
| ENR+RF+NB+DL | 0.7672 | 0.7625 | 0.7720 | 0.8065 | 0.8020 | 0.8110 | 0.7554 | 0.7492 | 0.7616 |
| GBM+ RF+NB+DL | 0.7672 | 0.7625 | 0.7720 | 0.8065 | 0.8020 | 0.8110 | 0.7554 | 0.7492 | 0.7616 |
| ENR+GBM+ RF+NB+DL | 0.7677 | 0.7630 | 0.7725 | 0.8070 | 0.8025 | 0.8114 | 0.7550 | 0.7488 | 0.7612 |

^a^ CI: confidence interval

^b^ DL: deep learning (neural networks)

^c^ ENR: elastic net regression

^d^ GBM: gradient boosting machine

^e^ NB: naive Bayes

^f^ RF: random forest

Supplementary Table 16. Specificity of machine learning models for predicting syphilis acquisition

| Models | Testing data  (2015-2018) | | | External validation data  (2019) | | | External validation data  (2020 - 2021) | | |
| --- | --- | --- | --- | --- | --- | --- | --- | --- | --- |
|  | Mean | 95%CI | | Mean | 95%CI | | Mean | 95%CI | |
| ENR | 0.7854 | 0.7525 | 0.8183 | 0.7395 | 0.7041 | 0.7749 | 0.7160 | 0.7096 | 0.7225 |
| GBM | 0.7595 | 0.7252 | 0.7939 | 0.7327 | 0.6968 | 0.7685 | 0.7433 | 0.7370 | 0.7496 |
| RF | 0.7253 | 0.6895 | 0.7610 | 0.7122 | 0.6756 | 0.7487 | 0.7360 | 0.7297 | 0.7423 |
| NB | 0.7978 | 0.7656 | 0.8301 | 0.7884 | 0.7554 | 0.8214 | 0.5549 | 0.5478 | 0.5620 |
| DL | 0.6424 | 0.6043 | 0.6806 | 0.5540 | 0.5142 | 0.5939 | 0.7748 | 0.7688 | 0.7808 |
| ENR+ GBM | 0.7770 | 0.7436 | 0.8103 | 0.7338 | 0.6980 | 0.7696 | 0.7337 | 0.7274 | 0.7400 |
| ENR +RF | 0.7644 | 0.7303 | 0.7984 | 0.7321 | 0.6962 | 0.7679 | 0.7373 | 0.7310 | 0.7436 |
| ENR + NB | 0.7854 | 0.7525 | 0.8183 | 0.7395 | 0.7041 | 0.7749 | 0.7160 | 0.7096 | 0.7225 |
| ENR + DL | 0.7718 | 0.7383 | 0.8054 | 0.7196 | 0.6834 | 0.7558 | 0.7374 | 0.7312 | 0.7437 |
| GBM + RF | 0.7408 | 0.7058 | 0.7759 | 0.7116 | 0.6749 | 0.7483 | 0.7493 | 0.7431 | 0.7555 |
| GBM + NB | 0.7563 | 0.7218 | 0.7907 | 0.7275 | 0.6915 | 0.7636 | 0.7435 | 0.7372 | 0.7497 |
| GBM + DL | 0.7548 | 0.7203 | 0.7893 | 0.7150 | 0.6785 | 0.7516 | 0.7548 | 0.7173 | 0.7922 |
| RF + NB | 0.7242 | 0.6884 | 0.7600 | 0.7110 | 0.6744 | 0.7476 | 0.7370 | 0.6987 | 0.7753 |
| RF + DL | 0.7128 | 0.6766 | 0.7490 | 0.6832 | 0.6455 | 0.7208 | 0.7226 | 0.6836 | 0.7615 |
| NB + DL | 0.7063 | 0.6700 | 0.7427 | 0.6576 | 0.6196 | 0.6955 | 0.7020 | 0.6627 | 0.7413 |
| ENR+ GBM+ RF | 0.7643 | 0.7303 | 0.7984 | 0.7247 | 0.6885 | 0.7608 | 0.7653 | 0.7284 | 0.8022 |
| ENR+ GBM+ NB | 0.7770 | 0.7436 | 0.8103 | 0.7338 | 0.6980 | 0.7696 | 0.7724 | 0.7359 | 0.8089 |
| ENR+GBM+ DL | 0.7700 | 0.7362 | 0.8037 | 0.7230 | 0.6868 | 0.7592 | 0.7633 | 0.7263 | 0.8003 |
| ENR+ RF+ NB | 0.7644 | 0.7303 | 0.7984 | 0.7319 | 0.6961 | 0.7678 | 0.7712 | 0.7346 | 0.8078 |
| ENR+ RF+ DL | 0.7559 | 0.7214 | 0.7903 | 0.7082 | 0.6714 | 0.7450 | 0.7488 | 0.7111 | 0.7866 |
| ENR+ NB + DL | 0.7718 | 0.7383 | 0.8054 | 0.7196 | 0.6834 | 0.7558 | 0.7579 | 0.7208 | 0.7951 |
| GBM+ RF + NB | 0.7408 | 0.7058 | 0.7759 | 0.7110 | 0.6744 | 0.7477 | 0.7561 | 0.7188 | 0.7934 |
| GBM + RF + DL | 0.7334 | 0.6980 | 0.7688 | 0.7036 | 0.6667 | 0.7406 | 0.7423 | 0.7042 | 0.7803 |
| GBM + NB + DL | 0.7516 | 0.7169 | 0.7862 | 0.7173 | 0.6808 | 0.7537 | 0.7541 | 0.7166 | 0.7916 |
| RF + NB +DL | 0.7126 | 0.6764 | 0.7489 | 0.6832 | 0.6455 | 0.7208 | 0.7212 | 0.6823 | 0.7602 |
| ENR+GBM+ RF+NB | 0.7643 | 0.7303 | 0.7984 | 0.7247 | 0.6885 | 0.7608 | 0.7653 | 0.7284 | 0.8022 |
| ENR+GBM+ RF+DL | 0.7569 | 0.7225 | 0.7913 | 0.7133 | 0.6767 | 0.7499 | 0.7574 | 0.7201 | 0.7947 |
| ENR+GBM+NB+DL | 0.7700 | 0.7362 | 0.8037 | 0.7230 | 0.6868 | 0.7592 | 0.7633 | 0.7263 | 0.8003 |
| ENR+RF+NB+DL | 0.7559 | 0.7214 | 0.7903 | 0.7082 | 0.6714 | 0.7450 | 0.7488 | 0.7111 | 0.7866 |
| GBM+ RF+NB+DL | 0.7559 | 0.7214 | 0.7903 | 0.7082 | 0.6714 | 0.7450 | 0.7488 | 0.7111 | 0.7866 |
| ENR+GBM+ RF+NB+DL | 0.7569 | 0.7225 | 0.7913 | 0.7133 | 0.6767 | 0.7499 | 0.7574 | 0.7201 | 0.7947 |

^a^ CI: confidence interval

^b^ DL: deep learning (neural networks)

^c^ ENR: elastic net regression

^d^ GBM: gradient boosting machine

^e^ NB: naive Bayes

^f^ RF: random forest

Supplementary Table 17. AUC of machine learning models for predicting gonorrhoea acquisition

| Models | Testing data  (2015-2018) | | | External validation data  (2019) | | | External validation data  (2020 - 2021) | | |
| --- | --- | --- | --- | --- | --- | --- | --- | --- | --- |
|  | Mean | 95%CI | | Mean | 95%CI | | Mean | 95%CI | |
| ENR | 0.7656 | 0.7567 | 0.7744 | 0.8031 | 0.7950 | 0.8112 | 0.7861 | 0.7757 | 0.7964 |
| GBM | 0.7773 | 0.7689 | 0.7857 | 0.8105 | 0.8029 | 0.8182 | 0.7915 | 0.7815 | 0.8015 |
| RF | 0.7701 | 0.7615 | 0.7786 | 0.8004 | 0.7926 | 0.8083 | 0.7767 | 0.7662 | 0.7871 |
| NB | 0.7311 | 0.7217 | 0.7405 | 0.7698 | 0.7609 | 0.7787 | 0.7501 | 0.7389 | 0.7613 |
| DL | 0.7626 | 0.7539 | 0.7713 | 0.7986 | 0.7906 | 0.8066 | 0.7795 | 0.7690 | 0.7900 |
| ENR+ GBM | 0.7780 | 0.7695 | 0.7864 | 0.8115 | 0.8039 | 0.8192 | 0.7928 | 0.7828 | 0.8028 |
| ENR +RF | 0.7758 | 0.7673 | 0.7843 | 0.8082 | 0.8004 | 0.8159 | 0.7876 | 0.7774 | 0.7978 |
| ENR + NB | 0.7656 | 0.7567 | 0.7744 | 0.8031 | 0.7950 | 0.8112 | 0.7861 | 0.7757 | 0.7964 |
| ENR + DL | 0.7657 | 0.7568 | 0.7746 | 0.8034 | 0.7953 | 0.8114 | 0.7862 | 0.7758 | 0.7965 |
| GBM + RF | 0.7782 | 0.7698 | 0.7866 | 0.8102 | 0.8025 | 0.8179 | 0.7897 | 0.7796 | 0.7998 |
| GBM + NB | 0.7773 | 0.7689 | 0.7857 | 0.8105 | 0.8028 | 0.8181 | 0.7915 | 0.7815 | 0.8015 |
| GBM + DL | 0.7773 | 0.7689 | 0.7857 | 0.8106 | 0.8029 | 0.8182 | 0.7915 | 0.7815 | 0.8016 |
| RF + NB | 0.7709 | 0.7624 | 0.7795 | 0.8006 | 0.7928 | 0.8085 | 0.7777 | 0.7673 | 0.7881 |
| RF + DL | 0.7701 | 0.7615 | 0.7787 | 0.8005 | 0.7926 | 0.8084 | 0.7767 | 0.7663 | 0.7872 |
| NB + DL | 0.7480 | 0.7393 | 0.7566 | 0.7868 | 0.7790 | 0.7946 | 0.7655 | 0.7551 | 0.7758 |
| ENR+ GBM+ RF | 0.7786 | 0.7702 | 0.7871 | 0.8107 | 0.8031 | 0.8184 | 0.7909 | 0.7808 | 0.8010 |
| ENR+ GBM+ NB | 0.7779 | 0.7695 | 0.7864 | 0.8115 | 0.8039 | 0.8192 | 0.7928 | 0.7828 | 0.8029 |
| ENR+GBM+ DL | 0.7779 | 0.7695 | 0.7864 | 0.8116 | 0.8039 | 0.8192 | 0.7929 | 0.7828 | 0.8029 |
| ENR+ RF+ NB | 0.7758 | 0.7673 | 0.7843 | 0.8082 | 0.8004 | 0.8159 | 0.7876 | 0.7774 | 0.7978 |
| ENR+ RF+ DL | 0.7758 | 0.7673 | 0.7843 | 0.8082 | 0.8005 | 0.8159 | 0.7876 | 0.7774 | 0.7978 |
| ENR+ NB + DL | 0.7657 | 0.7568 | 0.7746 | 0.8034 | 0.7953 | 0.8115 | 0.7862 | 0.7758 | 0.7965 |
| GBM+ RF + NB | 0.7782 | 0.7698 | 0.7866 | 0.8102 | 0.8025 | 0.8179 | 0.7897 | 0.7796 | 0.7998 |
| GBM + RF + DL | 0.7782 | 0.7698 | 0.7866 | 0.8102 | 0.8026 | 0.8179 | 0.7897 | 0.7796 | 0.7998 |
| GBM + NB + DL | 0.7773 | 0.7689 | 0.7857 | 0.8105 | 0.8028 | 0.8181 | 0.7915 | 0.7815 | 0.8016 |
| RF + NB +DL | 0.7710 | 0.7624 | 0.7795 | 0.8007 | 0.7928 | 0.8086 | 0.7777 | 0.7673 | 0.7882 |
| ENR+GBM+ RF+NB | 0.7786 | 0.7702 | 0.7871 | 0.8107 | 0.8031 | 0.8184 | 0.7909 | 0.7808 | 0.8010 |
| ENR+GBM+ RF+DL | 0.7786 | 0.7702 | 0.7871 | 0.8107 | 0.8031 | 0.8184 | 0.7909 | 0.7808 | 0.8010 |
| ENR+GBM+NB+DL | 0.7779 | 0.7695 | 0.7863 | 0.8116 | 0.8039 | 0.8192 | 0.7929 | 0.7828 | 0.8029 |
| ENR+RF+NB+DL | 0.7758 | 0.7673 | 0.7843 | 0.8082 | 0.8005 | 0.8160 | 0.7876 | 0.7774 | 0.7978 |
| GBM+ RF+NB+DL | 0.7758 | 0.7673 | 0.7843 | 0.8082 | 0.8005 | 0.8160 | 0.7876 | 0.7774 | 0.7978 |
| ENR+GBM+ RF+NB+DL | 0.7786 | 0.7702 | 0.7871 | 0.8107 | 0.8031 | 0.8184 | 0.7909 | 0.7808 | 0.8010 |

^a^ AUC: areas under the curve

^b^ CI: confidence interval

^c^ DL: deep learning (neural networks)

^d^ ENR: elastic net regression

^e^ GBM: gradient boosting machine

^f^ NB: naive Bayes

^g^ RF: random forest

Supplementary Table 18. Sensitivity of machine learning models for predicting gonorrhoea acquisition

| Models | Testing data  (2015-2018) | | | External validation data  (2019) | | | External validation data  (2020 - 2021) | | |
| --- | --- | --- | --- | --- | --- | --- | --- | --- | --- |
|  | Mean | 95%CI | | Mean | 95%CI | | Mean | 95%CI | |
| ENR | 0.6098 | 0.6043 | 0.6154 | 0.6901 | 0.6852 | 0.6950 | 0.6438 | 0.6373 | 0.6502 |
| GBM | 0.6452 | 0.6398 | 0.6506 | 0.7160 | 0.7113 | 0.7208 | 0.6695 | 0.6632 | 0.6758 |
| RF | 0.7145 | 0.7094 | 0.7196 | 0.7867 | 0.7824 | 0.7910 | 0.7441 | 0.7383 | 0.7500 |
| NB | 0.5767 | 0.5711 | 0.5823 | 0.7230 | 0.7183 | 0.7277 | 0.6485 | 0.6421 | 0.6549 |
| DL | 0.6175 | 0.6120 | 0.6230 | 0.7023 | 0.6975 | 0.7072 | 0.6535 | 0.6471 | 0.6599 |
| ENR+ GBM | 0.6414 | 0.6359 | 0.6468 | 0.7135 | 0.7087 | 0.7183 | 0.6665 | 0.6602 | 0.6728 |
| ENR +RF | 0.6889 | 0.6837 | 0.6941 | 0.7674 | 0.7629 | 0.7718 | 0.7223 | 0.7163 | 0.7283 |
| ENR + NB | 0.6098 | 0.6043 | 0.6154 | 0.6901 | 0.6852 | 0.6950 | 0.6438 | 0.6373 | 0.6502 |
| ENR + DL | 0.6099 | 0.6043 | 0.6154 | 0.6902 | 0.6853 | 0.6951 | 0.6439 | 0.6375 | 0.6503 |
| GBM + RF | 0.6879 | 0.6827 | 0.6931 | 0.7637 | 0.7592 | 0.7682 | 0.7223 | 0.7163 | 0.7283 |
| GBM + NB | 0.6450 | 0.6396 | 0.6504 | 0.7162 | 0.7114 | 0.7209 | 0.6693 | 0.6630 | 0.6757 |
| GBM + DL | 0.6451 | 0.6397 | 0.6505 | 0.7160 | 0.7112 | 0.7207 | 0.6694 | 0.6631 | 0.6757 |
| RF + NB | 0.7089 | 0.7038 | 0.7140 | 0.7825 | 0.7782 | 0.7869 | 0.7385 | 0.7326 | 0.7444 |
| RF + DL | 0.7144 | 0.7093 | 0.7195 | 0.7867 | 0.7823 | 0.7910 | 0.7440 | 0.7382 | 0.7499 |
| NB + DL | 0.5996 | 0.5941 | 0.6052 | 0.6999 | 0.6951 | 0.7047 | 0.6379 | 0.6314 | 0.6443 |
| ENR+ GBM+ RF | 0.6851 | 0.6799 | 0.6904 | 0.7601 | 0.7556 | 0.7646 | 0.7190 | 0.7129 | 0.7250 |
| ENR+ GBM+ NB | 0.6414 | 0.6360 | 0.6468 | 0.7133 | 0.7086 | 0.7181 | 0.6664 | 0.6601 | 0.6727 |
| ENR+GBM+ DL | 0.6413 | 0.6359 | 0.6467 | 0.7135 | 0.7087 | 0.7182 | 0.6664 | 0.6601 | 0.6728 |
| ENR+ RF+ NB | 0.6888 | 0.6836 | 0.6941 | 0.7673 | 0.7628 | 0.7718 | 0.7224 | 0.7164 | 0.7284 |
| ENR+ RF+ DL | 0.6890 | 0.6837 | 0.6942 | 0.7674 | 0.7629 | 0.7719 | 0.7224 | 0.7164 | 0.7284 |
| ENR+ NB + DL | 0.6099 | 0.6043 | 0.6154 | 0.6902 | 0.6853 | 0.6951 | 0.6439 | 0.6375 | 0.6503 |
| GBM+ RF + NB | 0.6879 | 0.6827 | 0.6932 | 0.7638 | 0.7593 | 0.7683 | 0.7223 | 0.7163 | 0.7283 |
| GBM + RF + DL | 0.6879 | 0.6826 | 0.6931 | 0.7637 | 0.7592 | 0.7682 | 0.7223 | 0.7163 | 0.7283 |
| GBM + NB + DL | 0.6449 | 0.6395 | 0.6503 | 0.7161 | 0.7113 | 0.7208 | 0.6692 | 0.6629 | 0.6755 |
| RF + NB +DL | 0.7089 | 0.7038 | 0.7141 | 0.7826 | 0.7783 | 0.7870 | 0.7385 | 0.7326 | 0.7444 |
| ENR+GBM+ RF+NB | 0.6850 | 0.6798 | 0.6903 | 0.7602 | 0.7556 | 0.7647 | 0.7189 | 0.7129 | 0.7249 |
| ENR+GBM+ RF+DL | 0.6852 | 0.6799 | 0.6904 | 0.7601 | 0.7556 | 0.7646 | 0.7190 | 0.7129 | 0.7250 |
| ENR+GBM+NB+DL | 0.6414 | 0.6359 | 0.6468 | 0.7133 | 0.7086 | 0.7181 | 0.6664 | 0.6601 | 0.6727 |
| ENR+RF+NB+DL | 0.6889 | 0.6837 | 0.6941 | 0.7673 | 0.7628 | 0.7718 | 0.7224 | 0.7164 | 0.7284 |
| GBM+ RF+NB+DL | 0.6889 | 0.6837 | 0.6941 | 0.7673 | 0.7628 | 0.7718 | 0.7224 | 0.7164 | 0.7284 |
| ENR+GBM+ RF+NB+DL | 0.6850 | 0.6798 | 0.6903 | 0.7601 | 0.7556 | 0.7646 | 0.7189 | 0.7129 | 0.7250 |

^a^ CI: confidence interval

^b^ DL: deep learning (neural networks)

^c^ ENR: elastic net regression

^d^ GBM: gradient boosting machine

^e^ NB: naive Bayes

^f^ RF: random forest

Supplementary Table 19. Specificity of machine learning models for predicting gonorrhoea acquisition

| Models | Testing data  (2015-2018) | | | External validation data  (2019) | | | External validation data  (2020 - 2021) | | |
| --- | --- | --- | --- | --- | --- | --- | --- | --- | --- |
|  | Mean | 95%CI | | Mean | 95%CI | | Mean | 95%CI | |
| ENR | 0.7921 | 0.7763 | 0.8080 | 0.7897 | 0.7731 | 0.8063 | 0.6438 | 0.6373 | 0.6502 |
| GBM | 0.7734 | 0.7571 | 0.7898 | 0.7589 | 0.7414 | 0.7763 | 0.6695 | 0.6632 | 0.6758 |
| RF | 0.6789 | 0.6608 | 0.6969 | 0.6298 | 0.6101 | 0.6494 | 0.7441 | 0.7383 | 0.7500 |
| NB | 0.8000 | 0.7844 | 0.8156 | 0.7079 | 0.6893 | 0.7265 | 0.6485 | 0.6421 | 0.6549 |
| DL | 0.7809 | 0.7648 | 0.7970 | 0.7634 | 0.7460 | 0.7807 | 0.6535 | 0.6471 | 0.6599 |
| ENR+ GBM | 0.7793 | 0.7631 | 0.7955 | 0.7661 | 0.7488 | 0.7834 | 0.6665 | 0.6602 | 0.6728 |
| ENR +RF | 0.7222 | 0.7047 | 0.7396 | 0.6837 | 0.6648 | 0.7027 | 0.7223 | 0.7163 | 0.7283 |
| ENR + NB | 0.7921 | 0.7763 | 0.8080 | 0.7897 | 0.7731 | 0.8063 | 0.6438 | 0.6373 | 0.6502 |
| ENR + DL | 0.7921 | 0.7763 | 0.8080 | 0.7897 | 0.7731 | 0.8063 | 0.6439 | 0.6375 | 0.6503 |
| GBM + RF | 0.7287 | 0.7114 | 0.7460 | 0.6875 | 0.6686 | 0.7064 | 0.7223 | 0.7163 | 0.7283 |
| GBM + NB | 0.7736 | 0.7573 | 0.7899 | 0.7596 | 0.7422 | 0.7770 | 0.6693 | 0.6630 | 0.6757 |
| GBM + DL | 0.7736 | 0.7573 | 0.7899 | 0.7594 | 0.7419 | 0.7768 | 0.7842 | 0.7639 | 0.8046 |
| RF + NB | 0.6872 | 0.6692 | 0.7052 | 0.6392 | 0.6196 | 0.6587 | 0.6652 | 0.6419 | 0.6885 |
| RF + DL | 0.6789 | 0.6608 | 0.6970 | 0.6298 | 0.6101 | 0.6494 | 0.6511 | 0.6276 | 0.6746 |
| NB + DL | 0.7940 | 0.7783 | 0.8098 | 0.7598 | 0.7424 | 0.7773 | 0.7934 | 0.7734 | 0.8134 |
| ENR+ GBM+ RF | 0.7326 | 0.7154 | 0.7498 | 0.6956 | 0.6768 | 0.7144 | 0.7199 | 0.6977 | 0.7421 |
| ENR+ GBM+ NB | 0.7794 | 0.7633 | 0.7956 | 0.7665 | 0.7493 | 0.7838 | 0.7906 | 0.7705 | 0.8108 |
| ENR+GBM+ DL | 0.7793 | 0.7631 | 0.7955 | 0.7665 | 0.7492 | 0.7837 | 0.7906 | 0.7705 | 0.8108 |
| ENR+ RF+ NB | 0.7222 | 0.7047 | 0.7396 | 0.6837 | 0.6648 | 0.7027 | 0.7076 | 0.6850 | 0.7301 |
| ENR+ RF+ DL | 0.7222 | 0.7047 | 0.7396 | 0.6837 | 0.6648 | 0.7027 | 0.7073 | 0.6848 | 0.7299 |
| ENR+ NB + DL | 0.7921 | 0.7763 | 0.8080 | 0.7897 | 0.7731 | 0.8063 | 0.8128 | 0.7935 | 0.8321 |
| GBM+ RF + NB | 0.7285 | 0.7112 | 0.7458 | 0.6874 | 0.6684 | 0.7063 | 0.7129 | 0.6905 | 0.7353 |
| GBM + RF + DL | 0.7288 | 0.7115 | 0.7461 | 0.6875 | 0.6686 | 0.7064 | 0.7129 | 0.6905 | 0.7353 |
| GBM + NB + DL | 0.7737 | 0.7574 | 0.7900 | 0.7601 | 0.7427 | 0.7775 | 0.7849 | 0.7645 | 0.8052 |
| RF + NB +DL | 0.6872 | 0.6692 | 0.7052 | 0.6390 | 0.6194 | 0.6586 | 0.6650 | 0.6416 | 0.6883 |
| ENR+GBM+ RF+NB | 0.7330 | 0.7158 | 0.7502 | 0.6958 | 0.6770 | 0.7145 | 0.7199 | 0.6977 | 0.7421 |
| ENR+GBM+ RF+DL | 0.7326 | 0.7154 | 0.7498 | 0.6956 | 0.6768 | 0.7144 | 0.7199 | 0.6977 | 0.7421 |
| ENR+GBM+NB+DL | 0.7794 | 0.7633 | 0.7956 | 0.7669 | 0.7496 | 0.7842 | 0.7908 | 0.7707 | 0.8110 |
| ENR+RF+NB+DL | 0.7222 | 0.7047 | 0.7396 | 0.6837 | 0.6648 | 0.7027 | 0.7073 | 0.6848 | 0.7299 |
| GBM+ RF+NB+DL | 0.7222 | 0.7047 | 0.7396 | 0.6837 | 0.6648 | 0.7027 | 0.7073 | 0.6848 | 0.7299 |
| ENR+GBM+ RF+NB+DL | 0.7329 | 0.7156 | 0.7501 | 0.6958 | 0.6770 | 0.7145 | 0.7199 | 0.6977 | 0.7421 |

^a^ CI: confidence interval

^b^ DL: deep learning (neural networks)

^c^ ENR: elastic net regression

^d^ GBM: gradient boosting machine

^e^ NB: naive Bayes

^f^ RF: random forest

Supplementary Table 20. AUC of machine learning models for predicting chlamydia acquisition

| Models | Testing data  (2015-2018) | | | External validation data  (2019) | | | External validation data  (2020 - 2021) | | |
| --- | --- | --- | --- | --- | --- | --- | --- | --- | --- |
|  | Mean | 95%CI | | Mean | 95%CI | | Mean | 95%CI | |
| ENR | 0.6926 | 0.6834 | 0.7018 | 0.6845 | 0.6748 | 0.6941 | 0.6922 | 0.6805 | 0.7040 |
| GBM | 0.6962 | 0.6871 | 0.7052 | 0.6802 | 0.6707 | 0.6897 | 0.6788 | 0.6671 | 0.6905 |
| RF | 0.6955 | 0.6864 | 0.7046 | 0.6841 | 0.6746 | 0.6935 | 0.6731 | 0.6612 | 0.6849 |
| NB | 0.6504 | 0.6409 | 0.6600 | 0.6460 | 0.6362 | 0.6559 | 0.6492 | 0.6371 | 0.6613 |
| DL | 0.6845 | 0.6752 | 0.6937 | 0.6734 | 0.6638 | 0.6831 | 0.6727 | 0.6607 | 0.6846 |
| ENR+ GBM | 0.7026 | 0.6936 | 0.7116 | 0.6892 | 0.6798 | 0.6987 | 0.6902 | 0.6786 | 0.7018 |
| ENR +RF | 0.7012 | 0.6922 | 0.7103 | 0.6916 | 0.6822 | 0.7010 | 0.6867 | 0.6750 | 0.6985 |
| ENR + NB | 0.6926 | 0.6834 | 0.7018 | 0.6845 | 0.6748 | 0.6941 | 0.6922 | 0.6805 | 0.7040 |
| ENR + DL | 0.6926 | 0.6834 | 0.7018 | 0.6845 | 0.6748 | 0.6941 | 0.6922 | 0.6805 | 0.7040 |
| GBM + RF | 0.7006 | 0.6915 | 0.7096 | 0.6871 | 0.6777 | 0.6965 | 0.6808 | 0.6691 | 0.6925 |
| GBM + NB | 0.6979 | 0.6888 | 0.7069 | 0.6822 | 0.6727 | 0.6917 | 0.6810 | 0.6693 | 0.6927 |
| GBM + DL | 0.6962 | 0.6871 | 0.7053 | 0.6803 | 0.6708 | 0.6898 | 0.6788 | 0.6671 | 0.6905 |
| RF + NB | 0.6964 | 0.6873 | 0.7055 | 0.6854 | 0.6760 | 0.6948 | 0.6752 | 0.6634 | 0.6871 |
| RF + DL | 0.6955 | 0.6864 | 0.7046 | 0.6841 | 0.6747 | 0.6935 | 0.6731 | 0.6612 | 0.6850 |
| NB + DL | 0.6609 | 0.6516 | 0.6701 | 0.6524 | 0.6427 | 0.6621 | 0.6518 | 0.6397 | 0.6638 |
| ENR+ GBM+ RF | 0.7036 | 0.6946 | 0.7126 | 0.6910 | 0.6816 | 0.7004 | 0.6879 | 0.6762 | 0.6995 |
| ENR+ GBM+ NB | 0.7026 | 0.6936 | 0.7116 | 0.6892 | 0.6798 | 0.6987 | 0.6902 | 0.6786 | 0.7018 |
| ENR+GBM+ DL | 0.7026 | 0.6936 | 0.7116 | 0.6892 | 0.6798 | 0.6987 | 0.6902 | 0.6786 | 0.7018 |
| ENR+ RF+ NB | 0.7012 | 0.6922 | 0.7103 | 0.6916 | 0.6823 | 0.7010 | 0.6868 | 0.6750 | 0.6985 |
| ENR+ RF+ DL | 0.7012 | 0.6922 | 0.7103 | 0.6916 | 0.6822 | 0.7010 | 0.6868 | 0.6750 | 0.6985 |
| ENR+ NB + DL | 0.6926 | 0.6834 | 0.7018 | 0.6845 | 0.6748 | 0.6941 | 0.6922 | 0.6805 | 0.7040 |
| GBM+ RF + NB | 0.7010 | 0.6920 | 0.7100 | 0.6875 | 0.6781 | 0.6969 | 0.6815 | 0.6698 | 0.6932 |
| GBM + RF + DL | 0.7006 | 0.6915 | 0.7096 | 0.6871 | 0.6777 | 0.6965 | 0.6808 | 0.6691 | 0.6925 |
| GBM + NB + DL | 0.6979 | 0.6888 | 0.7070 | 0.6822 | 0.6727 | 0.6917 | 0.6811 | 0.6694 | 0.6928 |
| RF + NB +DL | 0.6964 | 0.6873 | 0.7055 | 0.6854 | 0.6760 | 0.6949 | 0.6753 | 0.6634 | 0.6872 |
| ENR+GBM+ RF+NB | 0.7036 | 0.6946 | 0.7126 | 0.6910 | 0.6816 | 0.7004 | 0.6879 | 0.6762 | 0.6995 |
| ENR+GBM+ RF+DL | 0.7036 | 0.6946 | 0.7126 | 0.6910 | 0.6816 | 0.7004 | 0.6879 | 0.6762 | 0.6995 |
| ENR+GBM+NB+DL | 0.7026 | 0.6936 | 0.7116 | 0.6892 | 0.6798 | 0.6987 | 0.6902 | 0.6786 | 0.7018 |
| ENR+RF+NB+DL | 0.7012 | 0.6922 | 0.7103 | 0.6917 | 0.6823 | 0.7010 | 0.6868 | 0.6750 | 0.6985 |
| GBM+ RF+NB+DL | 0.7012 | 0.6922 | 0.7103 | 0.6917 | 0.6823 | 0.7010 | 0.6868 | 0.6750 | 0.6985 |
| ENR+GBM+ RF+NB+DL | 0.7036 | 0.6946 | 0.7126 | 0.6910 | 0.6816 | 0.7004 | 0.6879 | 0.6762 | 0.6995 |

^a^ AUC: areas under the curve

^b^ CI: confidence interval

^c^ DL: deep learning (neural networks)

^d^ ENR: elastic net regression

^e^ GBM: gradient boosting machine

^f^ NB: naive Bayes

^g^ RF: random forest

Supplementary Table 21. Sensitivity of machine learning models for predicting chlamydia acquisition

| Models | Testing data  (2015-2018) | | | External validation data  (2019) | | | External validation data  (2020 - 2021) | | |
| --- | --- | --- | --- | --- | --- | --- | --- | --- | --- |
|  | Mean | 95%CI | | Mean | 95%CI | | Mean | 95%CI | |
| ENR | 0.4512 | 0.4460 | 0.4564 | 0.4598 | 0.4544 | 0.4651 | 0.4330 | 0.4262 | 0.4398 |
| GBM | 0.5547 | 0.5495 | 0.5598 | 0.5653 | 0.5600 | 0.5706 | 0.5220 | 0.5152 | 0.5288 |
| RF | 0.5832 | 0.5781 | 0.5884 | 0.5918 | 0.5865 | 0.5970 | 0.5356 | 0.5288 | 0.5424 |
| NB | 0.3879 | 0.3828 | 0.3929 | 0.3898 | 0.3846 | 0.3951 | 0.3432 | 0.3367 | 0.3497 |
| DL | 0.4497 | 0.4446 | 0.4549 | 0.4542 | 0.4488 | 0.4595 | 0.4148 | 0.4081 | 0.4215 |
| ENR+ GBM | 0.5343 | 0.5291 | 0.5395 | 0.5454 | 0.5401 | 0.5508 | 0.5055 | 0.4987 | 0.5124 |
| ENR +RF | 0.5594 | 0.5542 | 0.5646 | 0.5698 | 0.5645 | 0.5751 | 0.5167 | 0.5099 | 0.5236 |
| ENR + NB | 0.4512 | 0.4460 | 0.4564 | 0.4598 | 0.4544 | 0.4651 | 0.4330 | 0.4262 | 0.4398 |
| ENR + DL | 0.4510 | 0.4458 | 0.4562 | 0.4596 | 0.4542 | 0.4649 | 0.4328 | 0.4260 | 0.4395 |
| GBM + RF | 0.5799 | 0.5748 | 0.5850 | 0.5908 | 0.5856 | 0.5961 | 0.5405 | 0.5337 | 0.5473 |
| GBM + NB | 0.5494 | 0.5442 | 0.5546 | 0.5602 | 0.5549 | 0.5655 | 0.5148 | 0.5080 | 0.5216 |
| GBM + DL | 0.5542 | 0.5491 | 0.5594 | 0.5647 | 0.5594 | 0.5700 | 0.5213 | 0.5145 | 0.5281 |
| RF + NB | 0.5781 | 0.5729 | 0.5832 | 0.5860 | 0.5807 | 0.5913 | 0.5274 | 0.5205 | 0.5342 |
| RF + DL | 0.5830 | 0.5779 | 0.5881 | 0.5916 | 0.5863 | 0.5969 | 0.5356 | 0.5288 | 0.5424 |
| NB + DL | 0.4229 | 0.4177 | 0.4280 | 0.4233 | 0.4180 | 0.4286 | 0.3623 | 0.3557 | 0.3689 |
| ENR+ GBM+ RF | 0.5669 | 0.5618 | 0.5721 | 0.5785 | 0.5732 | 0.5838 | 0.5290 | 0.5222 | 0.5358 |
| ENR+ GBM+ NB | 0.5342 | 0.5290 | 0.5394 | 0.5453 | 0.5400 | 0.5507 | 0.5054 | 0.4986 | 0.5122 |
| ENR+GBM+ DL | 0.5345 | 0.5293 | 0.5396 | 0.5455 | 0.5401 | 0.5508 | 0.5057 | 0.4988 | 0.5125 |
| ENR+ RF+ NB | 0.5594 | 0.5542 | 0.5646 | 0.5697 | 0.5644 | 0.5751 | 0.5168 | 0.5099 | 0.5236 |
| ENR+ RF+ DL | 0.5594 | 0.5543 | 0.5646 | 0.5699 | 0.5646 | 0.5753 | 0.5168 | 0.5100 | 0.5237 |
| ENR+ NB + DL | 0.4511 | 0.4459 | 0.4563 | 0.4596 | 0.4543 | 0.4650 | 0.4328 | 0.4260 | 0.4396 |
| GBM+ RF + NB | 0.5775 | 0.5723 | 0.5826 | 0.5884 | 0.5831 | 0.5937 | 0.5369 | 0.5301 | 0.5437 |
| GBM + RF + DL | 0.5799 | 0.5748 | 0.5850 | 0.5908 | 0.5856 | 0.5961 | 0.5405 | 0.5337 | 0.5473 |
| GBM + NB + DL | 0.5493 | 0.5441 | 0.5544 | 0.5602 | 0.5549 | 0.5655 | 0.5149 | 0.5081 | 0.5217 |
| RF + NB +DL | 0.5780 | 0.5728 | 0.5831 | 0.5859 | 0.5806 | 0.5912 | 0.5275 | 0.5206 | 0.5343 |
| ENR+GBM+ RF+NB | 0.5669 | 0.5618 | 0.5721 | 0.5786 | 0.5733 | 0.5839 | 0.5291 | 0.5222 | 0.5359 |
| ENR+GBM+ RF+DL | 0.5669 | 0.5618 | 0.5721 | 0.5786 | 0.5733 | 0.5839 | 0.5291 | 0.5223 | 0.5360 |
| ENR+GBM+NB+DL | 0.5344 | 0.5292 | 0.5396 | 0.5455 | 0.5401 | 0.5508 | 0.5057 | 0.4988 | 0.5125 |
| ENR+RF+NB+DL | 0.5598 | 0.5546 | 0.5650 | 0.5703 | 0.5650 | 0.5756 | 0.5171 | 0.5102 | 0.5239 |
| GBM+ RF+NB+DL | 0.5598 | 0.5546 | 0.5650 | 0.5703 | 0.5650 | 0.5756 | 0.5171 | 0.5102 | 0.5239 |
| ENR+GBM+ RF+NB+DL | 0.5669 | 0.5618 | 0.5721 | 0.5786 | 0.5733 | 0.5839 | 0.5291 | 0.5223 | 0.5359 |

^a^ CI: confidence interval

^b^ DL: deep learning (neural networks)

^c^ ENR: elastic net regression

^d^ GBM: gradient boosting machine

^e^ NB: naive Bayes

^f^ RF: random forest

Supplementary Table 22. Specificity of machine learning models for predicting chlamydia acquisition

| Models | Testing data  (2015-2018) | | | External validation data  (2019) | | | External validation data  (2020 - 2021) | | |
| --- | --- | --- | --- | --- | --- | --- | --- | --- | --- |
|  | Mean | 95%CI | | Mean | 95%CI | | Mean | 95%CI | |
| ENR | 0.7955 | 0.7819 | 0.8090 | 0.7746 | 0.7602 | 0.7890 | 0.4330 | 0.4262 | 0.4398 |
| GBM | 0.7163 | 0.7014 | 0.7313 | 0.6880 | 0.6722 | 0.7038 | 0.5220 | 0.5152 | 0.5288 |
| RF | 0.6901 | 0.6746 | 0.7056 | 0.6757 | 0.6597 | 0.6918 | 0.5356 | 0.5288 | 0.5424 |
| NB | 0.7977 | 0.7842 | 0.8112 | 0.7936 | 0.7796 | 0.8075 | 0.3432 | 0.3367 | 0.3497 |
| DL | 0.7906 | 0.7770 | 0.8043 | 0.7676 | 0.7530 | 0.7821 | 0.4148 | 0.4081 | 0.4215 |
| ENR+ GBM | 0.7448 | 0.7302 | 0.7594 | 0.7195 | 0.7041 | 0.7349 | 0.5055 | 0.4987 | 0.5124 |
| ENR +RF | 0.7185 | 0.7034 | 0.7336 | 0.7059 | 0.6902 | 0.7216 | 0.5167 | 0.5099 | 0.5236 |
| ENR + NB | 0.7955 | 0.7819 | 0.8090 | 0.7746 | 0.7602 | 0.7890 | 0.4330 | 0.4262 | 0.4398 |
| ENR + DL | 0.7957 | 0.7822 | 0.8093 | 0.7748 | 0.7604 | 0.7892 | 0.4328 | 0.4260 | 0.4395 |
| GBM + RF | 0.6979 | 0.6825 | 0.7132 | 0.6768 | 0.6607 | 0.6928 | 0.5405 | 0.5337 | 0.5473 |
| GBM + NB | 0.7246 | 0.7097 | 0.7394 | 0.6958 | 0.6800 | 0.7115 | 0.5148 | 0.5080 | 0.5216 |
| GBM + DL | 0.7167 | 0.7018 | 0.7317 | 0.6884 | 0.6726 | 0.7042 | 0.7254 | 0.7067 | 0.7441 |
| RF + NB | 0.6971 | 0.6817 | 0.7125 | 0.6822 | 0.6662 | 0.6982 | 0.7197 | 0.7007 | 0.7387 |
| RF + DL | 0.6906 | 0.6751 | 0.7061 | 0.6759 | 0.6598 | 0.6920 | 0.7105 | 0.6913 | 0.7297 |
| NB + DL | 0.7921 | 0.7785 | 0.8057 | 0.7806 | 0.7664 | 0.7949 | 0.8305 | 0.8146 | 0.8464 |
| ENR+ GBM+ RF | 0.7161 | 0.7010 | 0.7312 | 0.6960 | 0.6801 | 0.7118 | 0.7356 | 0.7169 | 0.7543 |
| ENR+ GBM+ NB | 0.7451 | 0.7305 | 0.7597 | 0.7195 | 0.7041 | 0.7349 | 0.7550 | 0.7368 | 0.7732 |
| ENR+GBM+ DL | 0.7445 | 0.7299 | 0.7591 | 0.7194 | 0.7040 | 0.7348 | 0.7549 | 0.7367 | 0.7730 |
| ENR+ RF+ NB | 0.7186 | 0.7035 | 0.7337 | 0.7059 | 0.6902 | 0.7216 | 0.7447 | 0.7262 | 0.7632 |
| ENR+ RF+ DL | 0.7185 | 0.7034 | 0.7336 | 0.7059 | 0.6902 | 0.7216 | 0.7444 | 0.7259 | 0.7629 |
| ENR+ NB + DL | 0.7957 | 0.7822 | 0.8093 | 0.7747 | 0.7603 | 0.7891 | 0.8134 | 0.7969 | 0.8300 |
| GBM+ RF + NB | 0.7017 | 0.6864 | 0.7170 | 0.6800 | 0.6639 | 0.6960 | 0.7199 | 0.7009 | 0.7389 |
| GBM + RF + DL | 0.6982 | 0.6828 | 0.7135 | 0.6768 | 0.6607 | 0.6928 | 0.7163 | 0.6973 | 0.7354 |
| GBM + NB + DL | 0.7245 | 0.7097 | 0.7394 | 0.6958 | 0.6800 | 0.7115 | 0.7364 | 0.7178 | 0.7549 |
| RF + NB +DL | 0.6972 | 0.6818 | 0.7126 | 0.6827 | 0.6667 | 0.6987 | 0.7197 | 0.7007 | 0.7387 |
| ENR+GBM+ RF+NB | 0.7161 | 0.7010 | 0.7312 | 0.6961 | 0.6802 | 0.7119 | 0.7356 | 0.7169 | 0.7543 |
| ENR+GBM+ RF+DL | 0.7160 | 0.7009 | 0.7311 | 0.6960 | 0.6801 | 0.7118 | 0.7356 | 0.7169 | 0.7543 |
| ENR+GBM+NB+DL | 0.7447 | 0.7301 | 0.7593 | 0.7193 | 0.7038 | 0.7347 | 0.7549 | 0.7367 | 0.7730 |
| ENR+RF+NB+DL | 0.7183 | 0.7032 | 0.7334 | 0.7058 | 0.6901 | 0.7215 | 0.7444 | 0.7259 | 0.7629 |
| GBM+ RF+NB+DL | 0.7183 | 0.7032 | 0.7334 | 0.7058 | 0.6901 | 0.7215 | 0.7444 | 0.7259 | 0.7629 |
| ENR+GBM+ RF+NB+DL | 0.7160 | 0.7009 | 0.7311 | 0.6960 | 0.6801 | 0.7118 | 0.7356 | 0.7169 | 0.7543 |

^a^ CI: confidence interval

^b^ DL: deep learning (neural networks)

^c^ ENR: elastic net regression

^d^ GBM: gradient boosting machine

^e^ NB: naive Bayes

^f^ RF: random forest

Figure S3. The selection process of HIV/STI models. DL: deep learning (neural networks); ENR: elastic net regression; GBM: gradient boosting machine; NB: naive Bayes; RF: random forest.


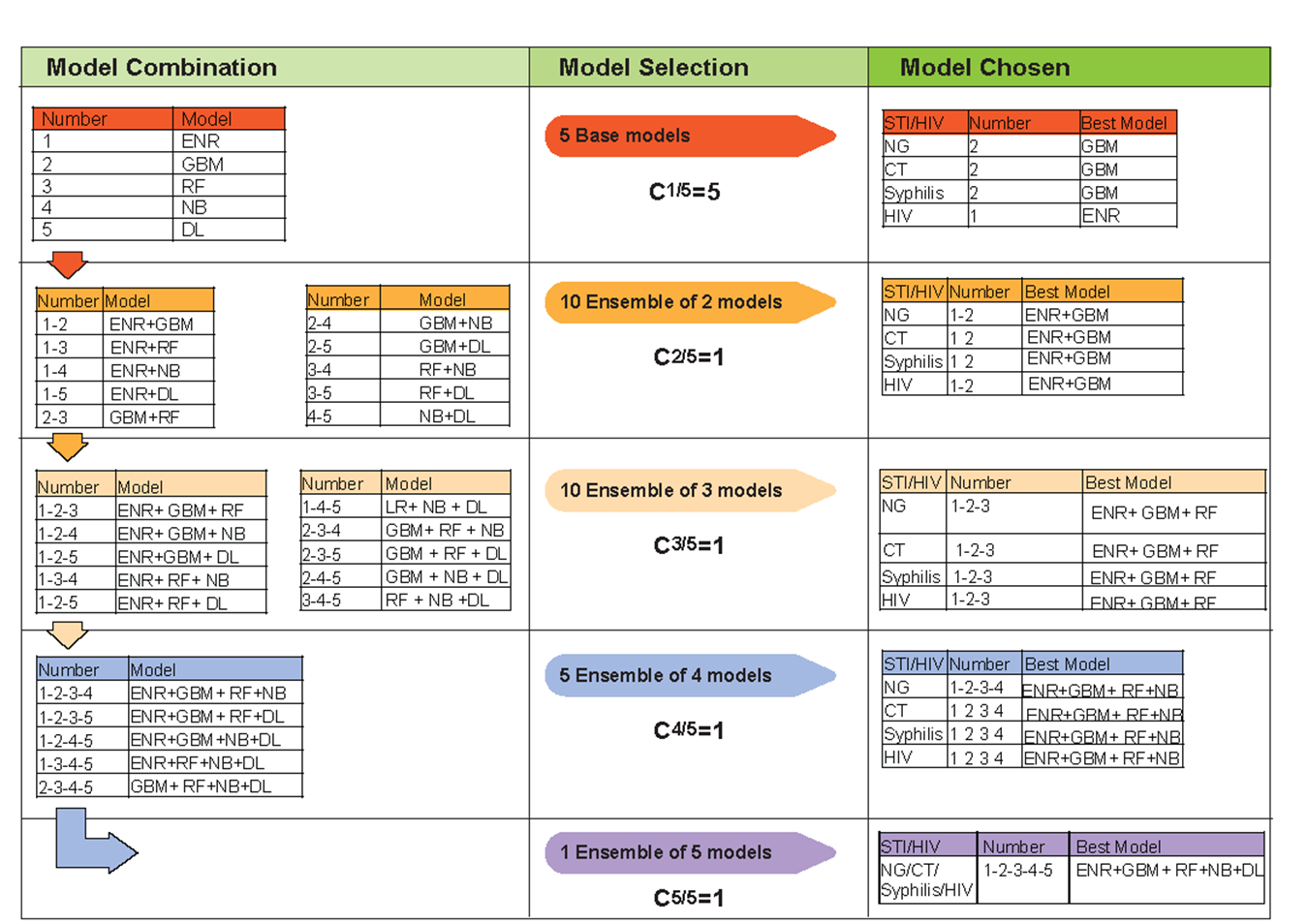


Development of a HIV/STI risk prediction tool

**Performance metrics of HIV/STI risk prediction tool**

Table S23. Performance metrics of HIV risk prediction tool

| Models | Testing data  (2015-2018) | | | External validation data  (2019) | | | External validation data  (2020 and January, 2021) | | |
| --- | --- | --- | --- | --- | --- | --- | --- | --- | --- |
|  | Mean | 95%CI | | Mean | 95%CI | | Mean | 95%CI | |
| AUC | 0.775 | 0.715 | 0.834 | 0.790 | 0.743 | 0.838 | 0.713 | 0.601 | 0.824 |
| Sensitivity | 0.731 | 0.725 | 0.738 | 0.696 | 0.691 | 0.702 | 0.659 | 0.652 | 0.666 |
| Specificity | 0.732 | 0.725 | 0.738 | 0.789 | 0.684 | 0.895 | 0.704 | 0.531 | 0.876 |

^a^ AUC: area under the receiver operating characteristic curve

^b^ CI: confidence interval

Table S24. Performance metrics of syphilis risk prediction tool

| Models | Testing data  (2015-2018) | | | External validation data  (2019) | | | External validation data  (2020 - 2021) | | |
| --- | --- | --- | --- | --- | --- | --- | --- | --- | --- |
|  | Mean | 95%CI | | Mean | 95%CI | | Mean | 95%CI | |
| AUC | 0.842 | 0.822 | 0.862 | 0.845 | 0.831 | 0.858 | 0.838 | 0.823 | 0.853 |
| Sensitivity | 0.753 | 0.747 | 0.759 | 0.782 | 0.777 | 0.787 | 0.713 | 0.706 | 0.719 |
| Specificity | 0.769 | 0.726 | 0.813 | 0.732 | 0.696 | 0.768 | 0.801 | 0.766 | 0.836 |

^a^ AUC: area under the receiver operating characteristic curve

^b^ CI: confidence interval

Table S25. Performance metrics of gonorrhoea risk prediction tool

| Models | Testing data  (2015-2018) | | | External validation data  (2019) | | | External validation data  (2020 - 2021) | | |
| --- | --- | --- | --- | --- | --- | --- | --- | --- | --- |
|  | Mean | 95%CI | | Mean | 95%CI | | Mean | 95%CI | |
| AUC | 0.776 | 0.765 | 0.787 | 0.806 | 0.798 | 0.814 | 0.790 | 0.780 | 0.800 |
| Sensitivity | 0.663 | 0.656 | 0.670 | 0.728 | 0.723 | 0.733 | 0.754 | 0.748 | 0.760 |
| Specificity | 0.753 | 0.731 | 0.774 | 0.731 | 0.713 | 0.749 | 0.662 | 0.639 | 0.685 |

^a^ AUC: area under the receiver operating characteristic curve

^b^ CI: confidence interval

Table S26. Performance metrics of chlamydia risk prediction tool

| Models | Testing data  (2015-2018) | | | External validation data  (2019) | | | External validation data  (2020 - 2021) | | |
| --- | --- | --- | --- | --- | --- | --- | --- | --- | --- |
|  | Mean | 95%CI | | Mean | 95%CI | | Mean | 95%CI | |
| AUC | 0.698 | 0.686 | 0.710 | 0.690 | 0.681 | 0.700 | 0.693 | 0.681 | 0.704 |
| Sensitivity | 0.494 | 0.487 | 0.501 | 0.483 | 0.478 | 0.489 | 0.439 | 0.432 | 0.446 |
| Specificity | 0.775 | 0.757 | 0.793 | 0.770 | 0.756 | 0.785 | 0.820 | 0.803 | 0.836 |

^a^ AUC: area under the receiver operating characteristic curve

^b^ CI: confidence interval

Table S27. AUC of the HIV/STI risk prediction tool and the best machine learning model using all predictors

|  | Testing data  (2015-2018) | External validation data  (2019) | External validation data  (2020-2021) |
| --- | --- | --- | --- |
|  | Mean/95%CI | Mean/95%CI | Mean/95%CI |
| **HIV** |  |  |  |
| Best ML | 0.78(0.74-0.83) | 0.80(0.75-0.84) | 0.70(0.61-0.80) |
| Risk assessment tool | 0.78(0.72-0.83) | 0.79(0.74-0.84) | 0.71(0.60-0.82) |
| **syphilis** |  |  |  |
| Best ML | 0.84(0.83-0.86) | 0.85(0.83-0.86) | 0.83(0.82-0.85) |
| Risk assessment tool | 0.84(0.82-0.86) | 0.85(0.83-0.86) | 0.84(0.82-0.85) |
| **gonorrhea** |  |  |  |
| Best ML | 0.78(0.77-0.79) | 0.81(0.80-0.82) | 0.79(0.78-0.80) |
| Risk assessment tool | 0.78(0.77-0.79) | 0.81(0.80-0.81) | 0.79(0.78-0.80) |
| **chlamydia** |  |  |  |
| Best ML | 0.70(0.69-0.71) | 0.69(0.68-0.70) | 0.69(0.68-0.70) |
| Risk assessment tool | 0.70(0.69-0.71) | 0.69(0.68-0.70) | 0.69(0.68-0.70) |

^a^ AUC: area under the receiver operating characteristic curve

^b^ CI: confidence interval

^c^ ML: machine learning

**Future HIV/STI risk estimates**

Figure S4. Calibration of HIV model-predicted probability to HIV prevalence.


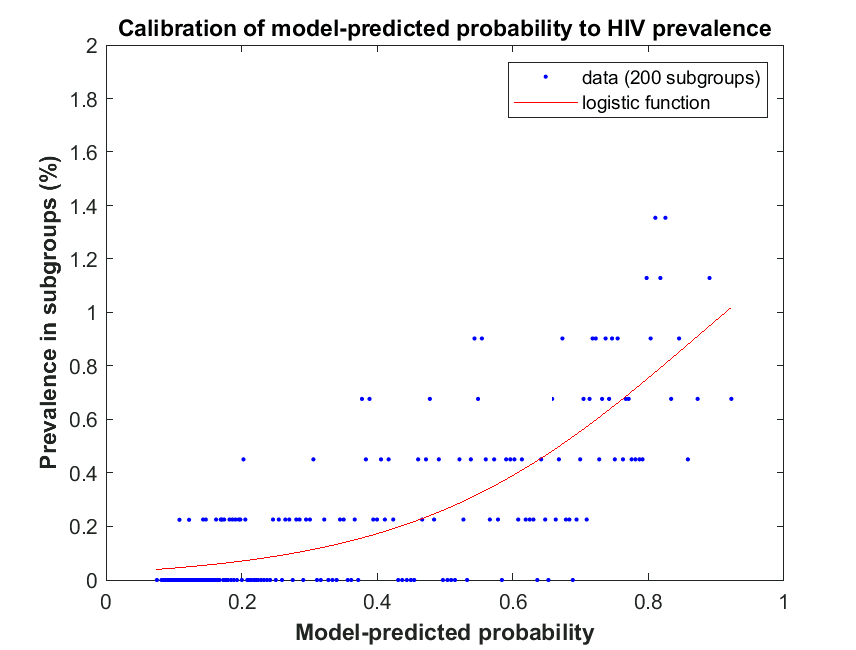


The logistic function assumes the form $\boldsymbol{f(x)=L/(1+}\boldsymbol{e}^{\boldsymbol{-r}\left（ \boldsymbol{x-}\boldsymbol{x}_{\boldsymbol{0}} \right）}\boldsymbol{)}$, where *L* = 1.820, *r* = 4.772 and *x_0_* = 0.872.

Figure S5. Calibration of syphilis model-predicted probability to syphilis prevalence.


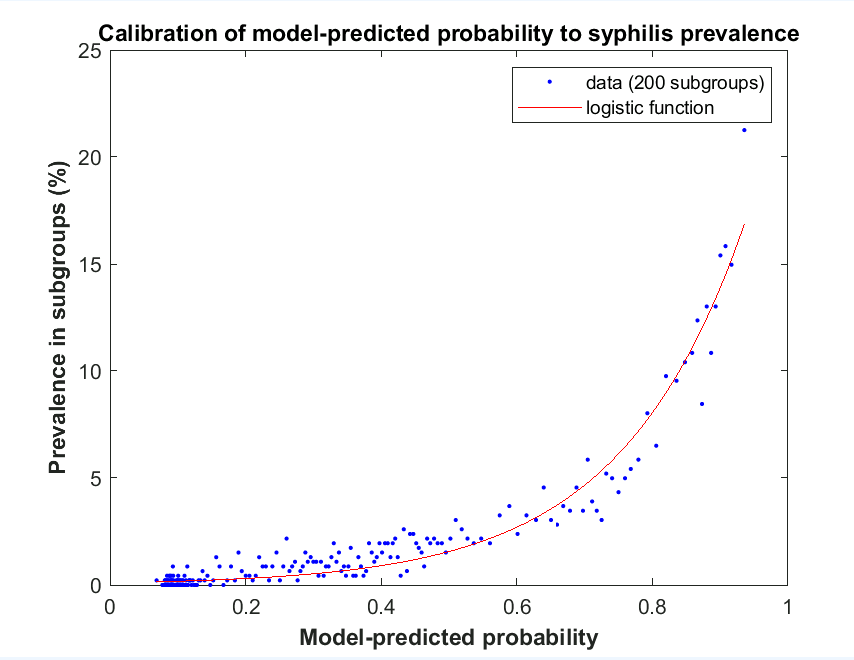


The logistic function assumes the form $\boldsymbol{f(x)=L/(1+}\boldsymbol{e}^{\boldsymbol{-r}\left（ \boldsymbol{x-}\boldsymbol{x}_{\boldsymbol{0}} \right）}\boldsymbol{)}$, where *L* = 100, *r* = 5.825 and *x_0_* = 1.215.

Figure S6. Calibration of gonorrhoea model-predicted probability to gonorrhoea prevalence.

**
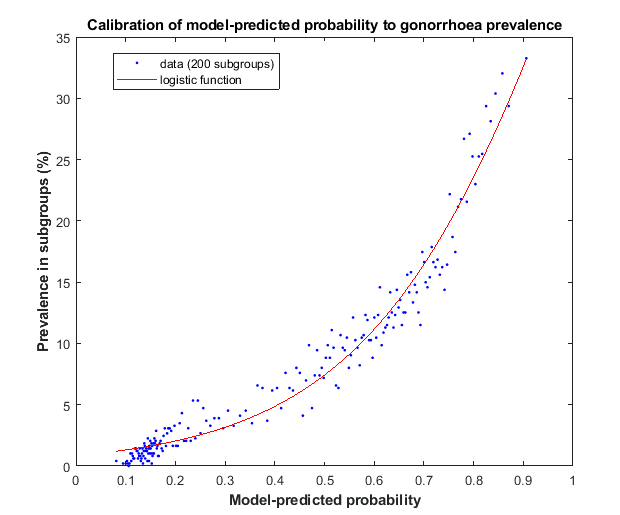
**

The logistic function assumes the form $\boldsymbol{f(x)=L/(1+}\boldsymbol{e}^{\boldsymbol{-r}\left（ \boldsymbol{x-}\boldsymbol{x}_{\boldsymbol{0}} \right）}\boldsymbol{)}$, where *L* = 100, *r* = 4.493 and *x_0_* = 1.062.

Figure S7. Calibration of chlamydia model-predicted probability to chlamydia prevalence.

**
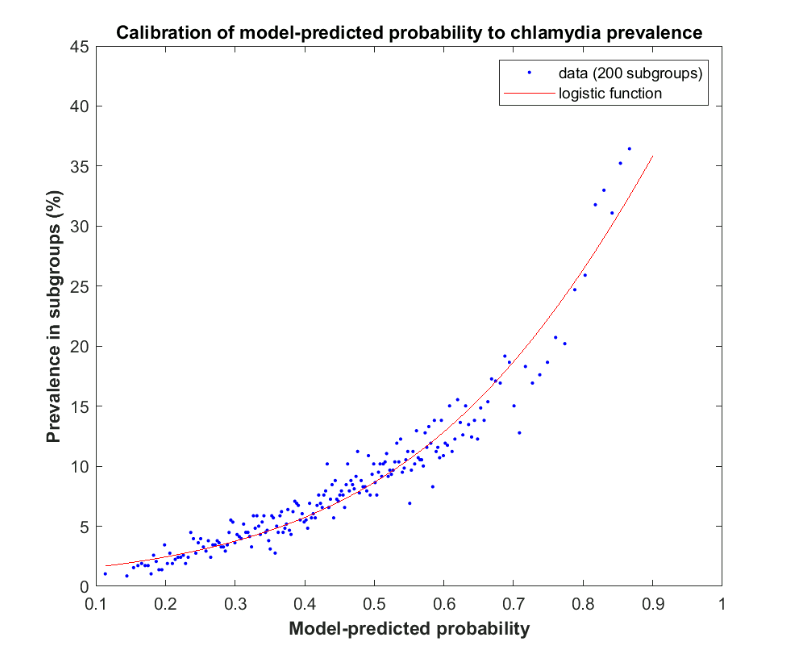
**

The logistic function assumes the form $\boldsymbol{f(x)=L/(1+}\boldsymbol{e}^{\boldsymbol{-r}\left（ \boldsymbol{x-}\boldsymbol{x}_{\boldsymbol{0}} \right）}\boldsymbol{)}$, where *L* = 100, *r* = 4.434 and *x_0_* = 1.031.

**HIV/STI risk prediction tool for screening**

Figure S8. HIV/STI risk prediction tool for screening in clinic websites or digital health platforms


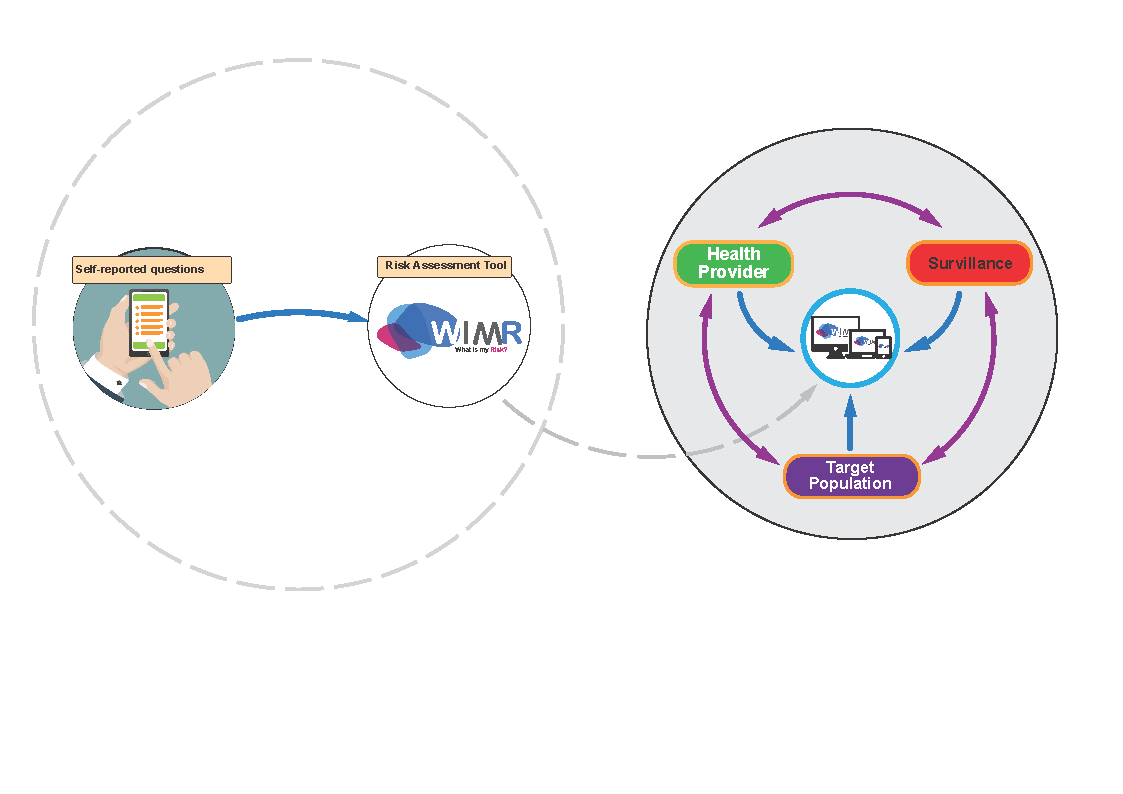

Supplement: Multimedia Appendix 1 [file jmir_v24i8e37850_app1.docx]
